# A streamlined CMR-derived machine-learning model for estimating cardiovascular biological age: development and validation in the UK-biobank and multi-ethnic study of atherosclerosis

**DOI:** 10.1093/ehjci/jeaf337

**Published:** 2025-12-04

**Authors:** Pier-Giorgio Masci, Gianni Andreozzi, Esther Puyol-Anton, Ashkan Abdollahi, Richard Mospan, Bram Ruijsink, Marina Cecelja, Phillip J Chowienczyk, Aqeel T Mohamed, Alistair Young, Bharath Ambale, Amedeo Chiribiri, Geoff Tison, Claire J Steves, Joao A C Lima, Reza Razavi, Valentina Lorenzoni, Andrew King

**Affiliations:** School of Biomedical Engineering and Imaging Sciences, King’s College London, London, UK; British Heart Foundation Centre of Excellence, King College London, London, UK; Institute of Management, Scuola Superiore Sant’Anna, Pisa, Italy; School of Biomedical Engineering and Imaging Sciences, King’s College London, London, UK; British Heart Foundation Centre of Excellence, King College London, London, UK; Division of Cardiology, John Hopkins University School of Medicine, Baltimore, MD, USA; School of Biomedical Engineering and Imaging Sciences, King’s College London, London, UK; School of Biomedical Engineering and Imaging Sciences, King’s College London, London, UK; British Heart Foundation Centre of Excellence, King College London, London, UK; Department of Twin Research and Genetic Epidemiology, School of Life Course and Population Sciences, King’s College London, London, UK; British Heart Foundation Centre of Excellence, King College London, London, UK; School of Cardiovascular Medicine & Metabolic Medicine & Sciences, King’s College London, London, UK; GKT School of Medical Education, King’s College London, London, UK; Faculty of Public Health and Policy, The London School of Hygiene and Tropical Medicine, London, UK; School of Biomedical Engineering and Imaging Sciences, King’s College London, London, UK; British Heart Foundation Centre of Excellence, King College London, London, UK; Department of Radiology, Johns Hopkins Hospital, Baltimore, MD, USA; School of Biomedical Engineering and Imaging Sciences, King’s College London, London, UK; British Heart Foundation Centre of Excellence, King College London, London, UK; Center for Biosignal Research, University of California, San Francisco, CA, USA; British Heart Foundation Centre of Excellence, King College London, London, UK; Department of Twin Research and Genetic Epidemiology, School of Life Course and Population Sciences, King’s College London, London, UK; Division of Cardiology, John Hopkins University School of Medicine, Baltimore, MD, USA; School of Biomedical Engineering and Imaging Sciences, King’s College London, London, UK; British Heart Foundation Centre of Excellence, King College London, London, UK; Institute of Management, Scuola Superiore Sant’Anna, Pisa, Italy; School of Biomedical Engineering and Imaging Sciences, King’s College London, London, UK; British Heart Foundation Centre of Excellence, King College London, London, UK

**Keywords:** ageing, biological age, cardiac magnetic resonance imaging, cardiovascular outcome

## Abstract

**Aims:**

Current models predicting cardiovascular biological age rely on radiomics or complex large feature sets including T1 and strain. We developed and validated a machine learning-based cardiovascular biological age estimate (*HeartAge*) using cardiovascular-magnetic-resonance (CMR) phenotypes and assessed the prognostic value of its deviation from chronological age (*HeartAge-gap*) for cardiovascular outcomes and mortality.

**Methods and results:**

*HeartAge* was developed using gradient-boosting regression in 3760 healthy UK-Biobank participants based on readily extractable CMR phenotypes. *HeartAge-gap* was defined as the difference between *HeartAge* and chronological age. The association of *HeartAge-gap* with prevalent cardiovascular conditions and composite cardiovascular outcome or all-cause mortality was tested in 31 784 UK-Biobank participants (64 ± 7 years; 16 640 females) and validated in 897 Multi-Ethnic Study of Atherosclerosis (MESA) participants (60 ± 10 years; 472 females) using logistic and Cox regression, respectively. Over a median 5.5-year follow-up (IQR: 4.7–7.1), 2316 (7.3%) and 363 (1.1%) participants experienced the composite cardiovascular outcome and all-cause mortality, respectively. Each one-year increase in *HeartAge-gap*, was associated with the composite cardiovascular outcome in females (HR: 1.022, 95% CI: 1.001–1.044, *P* = 0.048) and males (HR: 1.017, 95% CI: 1.002–1.033, *P* = 0.027) independently of chronological age and confounders including, body-mass-index, ischaemic heart disease, diabetes, and hypertension. In females only, increased *HeartAge-gap* predicted all-cause mortality (HR: 1.061, 95% CI: 1.007–1.118, *P* = 0.027), regardless of chronological age. In female MESA participants only, increased *HeartAge-gap* predicted the cardiovascular outcome (HR: 1.113, 95% CI: 1.025–1.210, *P* = 0.011) independently of chronological age and other confounders.

**Conclusion:**

A biologically older cardiovascular system was independently associated with adverse cardiovascular outcomes across both sexes. In females, advanced cardiovascular ageing also predicts all-cause mortality, irrespective of chronological age.

## Graphical Abstract

**Graphical Abstract:**
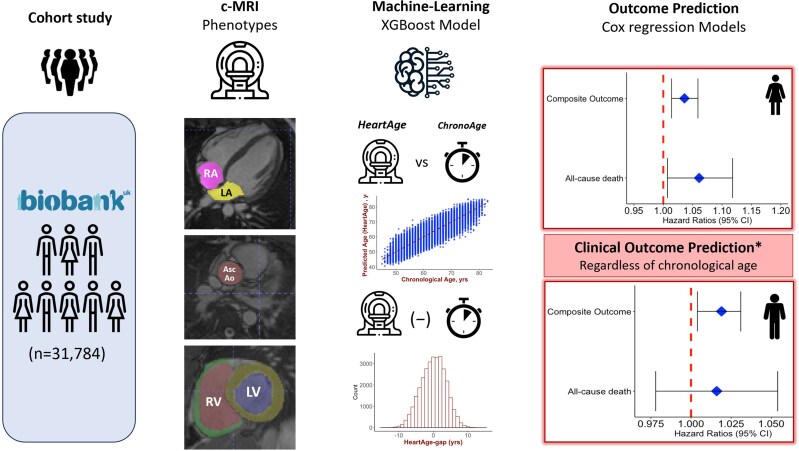
The figure outlines the main components of the study, and the clinical outcome prediction of the Cox regression *HeartAge* models. The composite cardiovascular outcome is an aggregate of cardiovascular death, ischaemic heart disease, ischaemic stroke, heart failure and cardiac rhythm abnormalities. *The results were externally tested and validated in the Multi-Ethnic Study of Atherosclerosis. Asc Ao, Ascending aorta; c-MRI, Cardiovascular Magnetic Resonance Imaging; LA, Left atrium; LV, Left ventricle; RA, Right atrium; RV, Right ventricle.

**See the editorial comment for this article ‘Imaging the aging heart', by S. Tahasildar and D.P. O’Regan, https://doi.org/10.1093/ehjci/jeaf354.**

## Introduction

Cardiovascular diseases are the leading cause of morbidity and mortality globally, with advanced age emerging as the principal risk factor.^1–3^ The most effective strategy to alleviate cardiovascular disease burden, and its societal and economic costs, consists in delaying disease onset and progression by directly addressing cardiovascular ageing.^4,5^

The major challenge in this paradigm lies in accurately determining an individual's ‘actual’ cardiovascular age, as chronological age poorly reflects the cardiovascular ageing processes.^2–5^ A promising approach consists in estimating the biological age of the cardiovascular system using machine-learning applied to a range of age-dependent cardiovascular phenotypes.^4–13^ Importantly, obtaining cardiovascular-specific biological ageing estimates are crucial as emerging evidence indicates that different organs within the same individual can follow distinct ageing trajectories.^14,15^

Cardiovascular magnetic resonance imaging (MRI) is uniquely suited to phenotype the cardiovascular system, offering an unparalleled accuracy and precision in quantifying structural and functional age-related changes.^12,13,16^ Although recent studies have explored the potential of machine-learning algorithms, informed by cardiovascular MRI phenotypes, for estimating cardiovascular biological age,^13,16^ their reliance on complex post-processing and advanced computational techniques limit their applicability. Moreover, these studies failed to validate cardiovascular biological ageing against clinical outcomes.

In this study, we aimed (i) develop a personalized estimate of cardiovascular biological age (*HeartAge*), and its deviation from chronological age (*HeartAge-gap*), by employing supervised machine-learning on readily extractable MRI phenotypes obtainable from a standard clinical scan; (ii) longitudinally validate *HeartAge-gap* against cardiovascular outcomes and all-cause mortality; (iii) externally validate its predictive performance in the Multi-Ethnic Study of Atherosclerosis (MESA) cohort.^17,18^

## Methods

### Sample

We utilized data from the UK-Biobank, a large population-based prospective cohort study of ∼500 000 individuals aged 40 and over. The UK-Biobank contains multidimensional data on health, lifestyle, physical and cognitive status, biological samples, genotype, and health outcomes. For our investigation, we focused on the sub-cohort of subjects who underwent cardiovascular MRI.^19^ We limited the analysis to individuals of European ancestry, given that the sample size was inadequate to build distinct models for other ancestries. This study complied with the UK-Biobank ethical approval from the National Health Service on 17 June 2021 (Ref: 11/NW/0382), and the subsequent extension on 18 June 2021 (Ref: 21/NW/0157).

### Cardiovascular magnetic resonance

The MRI protocol used in this study has been previously described.^12,13,16,19^ In brief, images were acquired using clinical wide bore 1.5-Tesla scanners (MAGNETOM Aera, Syngo Platform VD13A, Siemens Healthcare). The scan protocol consisted of cardiac short-axis, long-axis, and ascending aorta cine images. Structural and functional phenotypes of both right and left cardiac chambers, as well as the ascending aorta, were automatically obtained by our quality-controlled machine-learning pipeline.^20–22^

### Biological age of the cardiovascular system

**Figure 1:**
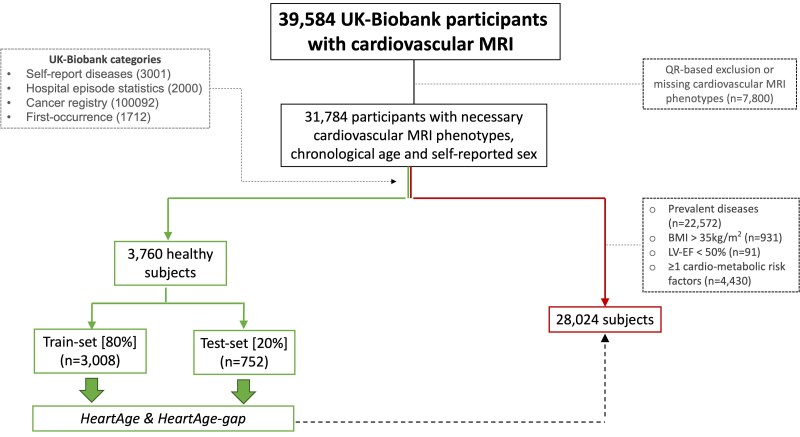
Study Flow chart. Healthy subjects were identified as those with no prevalent diseases, or cardiovascular or metabolic risk factors at the time of cardiovascular MRI, as indicated by self-report (UK-Biobank data-field 3001), hospital episode statistics (UK-Biobank data-field 2000), cancer registry (UK-Biobank data-field 100 092), and first-occurrence (UK-Biobank data-field 1712) categories. Once measured in the healthy subjects, *HeartAge* and *HeartAge-gap* were then also estimated in the 28 024 subjects with prevalent diseases/cardio-metabolic risk factors, morbid obesity (BMI > 35 kg/m^2^) or left ventricular ejection-fraction < 50%. BMI, Body-Mass-Index; LV-EF, Left ventricular ejection-fraction; MRI, Magnetic Resonance Imaging; QR, Quality-Review.

**Figure 2:**
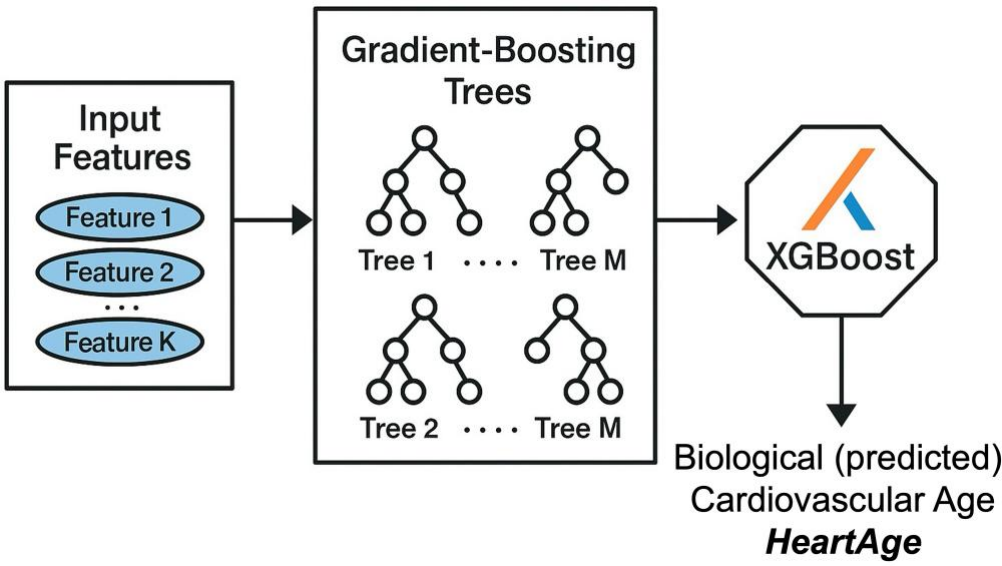
Schematic representation of *HeartAge* modelling using XGBoost regression. Readily extractable cardiovascular magnetic resonance (CMR) phenotypes serve as input features. These features are processed through an ensemble of gradient-boosting decision trees within the XGBoost algorithm to generate the predicted biological age of the cardiovascular system (*HeartAge*). The *HeartAge-gap* is defined as the difference between *HeartAge* and chronological age and is used in downstream analyses to evaluate associations with prevalent cardiovascular conditions (logistic regression modelling) and cardiovascular outcome and mortality (Cox regression modelling).

*Figure 1* outlines the study flow. In the imaging sub-cohort (*n* = 39 584), a total of 31 784 individuals had complete data for self-reported sex (UK-Biobank data-field 31), chronological age, and the cardiovascular MRI phenotypes needed to estimate biological age. Chronological age was calculated as the number of years from the year of birth (UK-Biobank data-field 34) to the cardiovascular MRI date (UK-Biobank data-field 53, instance 2). Within this subset, to derive the normative biological age of the cardiovascular system, we identified healthy subjects as those with body-mass-index (BMI) <35 kg/m^2^, left ventricular ejection-fraction >50%,^23^ no prevalent diseases, and no cardiovascular or metabolic risk factors at the time of cardiovascular MRI. These factors were determined based on self-report (UK-Biobank data-field 3001), hospital episode statistics (UK-Biobank data-field 2000), cancer registry (UK-Biobank data-field 100 092), and first-occurrence (UK-Biobank data-field 1712) categories (see Supplementary data online, *Table S1*). These healthy subjects [*n* = 3,760, 2008 (54%) female, median age 64 years, interquartile-range 58–69 years] were randomly divided into training (80%) and test (20%) sets. In the training-set, cardiovascular MRI phenotypes (*n* = 37; Supplementary data online, *Table S2*) and sex were introduced into the machine-learning gradient boosting regression model (XGBoost) to predict an individual's chronological age (*Figure 2*),^24^ The model underwent hyper-parametrisation using a 5-fold cross-validation algorithm, and SHapley Additive exPlanations analysis was applied to rank the most important features contributing to the model.^25^ The model's performance was subsequently assessed using the test-set (see Supplementary data online, Material). To mitigate regression dilution bias, predicted age was corrected using a well-validated statistical approach.^13,26^ The corrected predicted age, reflecting the biological age of the cardiovascular system (hereafter referred to as *HeartAge*), was also calculated for the UK-Biobank participants with prevalent diseases or cardio-metabolic risk factors (*n* = 28 024), (*Figure 1*). By subtracting an individual's *HeartAge* from their chronological age, we derived the *HeartAge-gap*. A *HeartAge-gap* >0 or <0 indicated an older or younger cardiovascular system relative to individuals of the same chronological age, respectively.^4–11^ Given the well-documented influence of sex on ageing trajectories,^4–13,16^ including differential patterns of cardiovascular remodelling and vascular ageing, analyses were conducted separately for males and females.

### Association of *HeartAge-gap* with cardiovascular conditions

A core concept of biological ageing suggests that deviation from chronological ageing, as measured by the *HeartAge-gap*, correlates with a higher likelihood of experiencing age-related conditions,^4–11^ irrespective of chronological age. Therefore, cross-sectional analyses were conducted to explore the relationship between *HeartAge-gap* and age-dependent cardiovascular conditions by developing logistic regression models, adjusted for chronological age, as detailed in the statistical analysis.

### *HeartAge-gap* and clinical outcome

The ability to predict clinical outcomes regardless of chronological age represents a key component in the validation of novel biological age metrics.^4–11^ Thus, we investigated the association of *HeartAge-gap* with a composite cardiovascular outcome aggregating cardiovascular death, ischaemic heart disease, ischaemic stroke, heart failure, and cardiac rhythm abnormalities (see Supplementary data online, *Table S3*). We also explored the association between *HeartAge-gap* and all-cause mortality. The ability of *HeartAge-gap* in predicting clinical outcomes was assessed by Cox regression proportional hazard-ratio models as detailed in the statistical analysis. ‘Time to event’ was measured as the number of days elapsed from the cardiovascular MRI date to the event. Dates and causes of death were obtained from the death register (UK-Biobank data-field 40 000), while the first-occurrence category (UK-Biobank data-field 1712) was used to identify other adverse events and their corresponding dates. Participants were censored at the time of the first occurring event, while those without events were censored at the last follow-up update (15 October 2023).

### Association of *HeartAge-gap* with cardiovascular phenotypes

To better investigate the sex-specific impact of ageing on the cardiovascular system, we assessed the relationship between *HeartAge-gap* and the main cardiovascular MRI phenotypes in males and females. Thus, we developed linear regression models, adjusted for chronological age and anthropometric measures, as detailed in the statistical analysis.

### Validation in the multi-ethnic study of atherosclerosis cohort

To assess the generalizability of our framework, we tested our study findings in the MESA cohort.^17,18^ Among the 6814 individuals aged between 45 and 84, recruited from July 2000 to August 2002, and belonging to different self-reported ethnic groups (Black, White, Hispanic/Latino and Chinese ethnicities), we identified 894 subjects [472 (53%) females, median age 61 years, interquartile-range 52–68 years] who had the necessary cardiovascular MRI phenotypes to estimate *HeartAge* (see Supplementary data online, *Table S4*). The XGBoost model was applied to the MESA cohort, and *HeartAge*(mesa) and *HeartAge-gap*(mesa) were measured as reported above (see Supplementary data online, *Methods*). Cox regression proportional hazard-ratio models were employed to assess the association of *HeartAge-gap*(mesa) with a hard cardiovascular outcome—a composite of incident myocardial infarction, ischaemic stroke and cardiovascular death (including resuscitated cardiac arrest and stroke death).

### Statistical analysis

Continuous variables were expressed as mean ± standard deviation (or median [interquartile range] if non-normally distributed) and categorical variables as *n* (%). Pearson’s correlation coefficient (*r*) was used to test the correlation between *HeartAge* and chorological age. Density plots visualized the distribution of chronological age, *HeartAge,* and *HeartAge-ga*p. Logistic regression assessed the association between *HeartAge-gap* and prevalent cardiovascular conditions. In the model, the cardiovascular condition was introduced as the dependent variable, while *HeartAge-gap*, chronological age, and squared chronological age (age^2^) were the independent variables.^12–14,16,27,28^ Cox regression proportional hazard-ratio models assessed the association between *HeartAge-gap* and clinical outcome. *Parsimonious models* included the composite cardiovascular outcome, its individual components, or all-cause mortality as the dependent variable, with *HeartAge-gap* and chronological age as independent variables. *Fully-adjusted models* incorporated *HeartAge-gap*, chronological age, BMI, diabetes, hypertension, and ischaemic heart disease at baseline as independent variables. In the MESA cohort, Cox regression models evaluated the association between *HeartAge-gap*(mesa) and cardiovascular outcome after adjusting for chronological age, hypertension, BMI and diabetes. Multivariable linear regression models tested the relationship between *HeartAge-gap* and cardiovascular MRI phenotypes where each MRI phenotype was introduced as the dependent variable, with *HeartAge-gap*, chronological-age, squared chronological age (age^2^), body weight (kg), and height (cm) being independent variables as previously reported.^12–14,16,27,28^ Model diagnostics confirmed the absence of significant multicollinearity (variance inflation factor < 5) and no evidence of heteroscedasticity, as assessed by inspection of residuals-vs.-fitted values plots for the multivariable linear regression models. For Cox regression models, the proportional hazards assumption was verified using scaled Schoenfeld residuals, with no violations observed. For all analyses, *P*-values <0.05 were deemed statistically significant. Analyses were carried out using the R software (R Statistical v4.1.2 using RStudio-IDE Version 2024.04.1 by Posit Software PBC).

## Results

### The biological age of the cardiovascular system

In the healthy subjects, the XGBoost model was trained on 3008 subjects (training-set) and tested in 752 subjects (test-set) resulting in Pearson’s correlation coefficient (*r*) of 0.914 (95% CI: 0.908–0.919, *P* < 0.001) and a mean absolute error of 9.05 years after correction for the regression to the mean bias.^16,26^ No correlation was observed between *HeartAge-gap* and chronological age (*P* = 0.999), indicating that deviation from normative cardiovascular ageing was unrelated to individuals’ chronological age. SHapley Additive exPlanations analysis revealed that aortic distensibility was the most critical feature in predicting *HeartAge*, as determined by the XGBoost model, followed by atrial and ventricular structural and functional phenotypes (see Supplementary data online, *Figure S1*). Finally, *HeartAge* and *HeartAge-gap* were successfully calculated in 28 024 participants with prevalent diseases or cardio-metabolic risk factors (*Figure 1*). These subjects were aggregated with the healthy subset to constitute our study cohort (*n* = 31 784), within which all further analyses were conducted separately for males and females. Baseline characteristics for the study cohort, stratified by sex, are outlined in *Table 1*.

**Table 1:** Baseline characteristics of the study cohort

	Female (*n* = 16 640)	Male (*n* = 15 144)
**Baseline characteristics**		
Age (years)	63.12 ± 7.36	64.45 ± 7.63
Body-mass-index (kg/m^2^)	25.82 ± 4.48	26.89 ± 3.86
Systolic blood pressure (mmHg)	136.89 ± 20.16	143.14 ± 18.29
Diastolic blood pressure (mmHg)	76.76 ± 10.45	80.51 ± 10.34
Heart rate (bpm)	70.43 ± 11.18	67.01 ± 12.19
Active smoking, *n* (%)	4831 (33.4%)	5388 (40.68%)
Diabetes, *n* (%)	485 (2.9%)	941 (6.2%)
Hypertension, *n* (%)	3285 (19.7%)	4618 (30.49%)
Dyslipidemia, *n* (%)	2649 (15.9%)	4003 (26.4%)
Ischaemic heart disease, *n* (%)	437 (2.6%)	1102 (7.2%)
Cardiac rhythm abnormalities, *n* (%)	557 (3.3%)	883 (5.8%)
**MRI Phenotypes**		
LV end-diastolic volume (mL)	131.99 ± 24.41	170.88 ± 34.66
LV end-systolic volume (mL)	43.11 ± 12.96	60.69 ± 20.07
LV stroke volume (mL)	88.88 ± 16.65	110.2 ± 22.44
LV ejection fraction (%)	67.59 ± 6.21	64.85 ± 7.01
LV end-diastolic mass (g)	78.63 ± 13.66	111.8 ± 20.02
RV end-diastolic volume (mL)	141.99 ± 28.45	188.31 ± 40.64
RV end-systolic volume (mL)	55.29 ± 14.26	78.02 ± 20.53
RV stroke volume (%)	86.7 ± 19.93	110.29 ± 27.35
RV ejection fraction (%)	60.99 ± 6.68	58.47 ± 6.77
RA reservoir volume (mL)	71.31 ± 18.87	96.74 ± 29.3
RA conduit volume (mL)	35.17 ± 12.09	50.39 ± 19.49
RA pump volume (mL)	21.19 ± 7.69	27.81 ± 11.01
LA reservoir volume (mL)	64.81 ± 18.34	75.54 ± 24.53
LA conduit volume (mL)	21.96 ± 10.96	27.09 ± 15.72
LA pump volume (mL)	20.1 ± 7.09	24.43 ± 8.76
Ascending aorta area at end-diastole (cm^2^)	7.93 ± 1.8	9.36 ± 2.08
Ascending aorta area at end-systole (cm^2^)	8.62 ± 1.74	10.19 ± 2.06
Ascending aorta distensibility (10^−3^ mmHg^−1^)	0.002 ± 0.001	0.002 ± 0.001
Basal anterior wall thickness (mm)	7.02 ± 1.02	8.2 ± 1.24
Basal anterior septum thickness (mm)	6.29 ± 1.39	7.5 ± 1.62
Basal inferior septum thickness (mm)	5.56 ± 1.19	6.61 ± 1.44
Basal inferior wall thickness (mm)	6.09 ± 0.81	7.07 ± 0.99
Basal inferolateral wall thickness (mm)	5.81 ± 0.75	6.73 ± 0.96
Basal anterolateral wall thickness (mm)	6.12 ± 0.77	7.11 ± 1.01
Mid anterior wall thickness (mm)	5.39 ± 0.57	6.27 ± 0.8
Mid anterior septum thickness (mm)	6.42 ± 0.8	7.7 ± 1
Mid inferior septum thickness (mm)	6.63 ± 0.94	8.18 ± 1.12
Mid inferior wall thickness (mm)	5.79 ± 0.73	6.89 ± 0.92
Mid inferolateral wall thickness (mm)	5.28 ± 0.63	6.24 ± 0.88
Mid anterolateral wall thickness (mm)	5.31 ± 0.52	6.21 ± 0.81
Apical anterior wall thickness (mm)	5.23 ± 0.62	5.74 ± 0.66
Apical septal thickness (mm)	5.47 ± 0.79	6.47 ± 0.86
Apical inferior wall thickness (mm)	4.6 ± 0.74	5.5 ± 0.81
Apical lateral wall thickness (mm)	4.93 ± 0.67	5.6 ± 0.75
Average LV wall thickness (mm)	5.81 ± 0.55	6.81 ± 0.7

LA, Left atrium; LV, Left ventricular; MRI, Magnetic Resonance Imaging; RA, Right atrium; RV, Right ventricular.

Atrial volumes: LA volumes were derived from bi-planar analysis of 2- and 4-chamber cine images, and RA volumes from 4-chamber cine only. Volumes were sampled at: (1) ventricular end-systole (maximum atrial volume, reservoir), (2) ventricular diastole immediately before atrial contraction (conduit), and (3) ventricular end-diastole after atrial contraction (minimum atrial volume, pump). Accordingly, reservoir volume = maximal atrial volume, conduit volume = atrial volume pre-atrial contraction, and pump volume = minimal atrial volume.

Ascending Aortic Distensibility (AoD): The cross-sectional area of the ascending aorta was extracted across the cardiac cycle, with minimum (Amin) and maximum (Amax) areas identified. Distensibility was computed as (Amax − Amin) / (Amin × PP), where PP denotes central pulse pressure in mmHg measured at the time of MRI.

**Figure 3:**
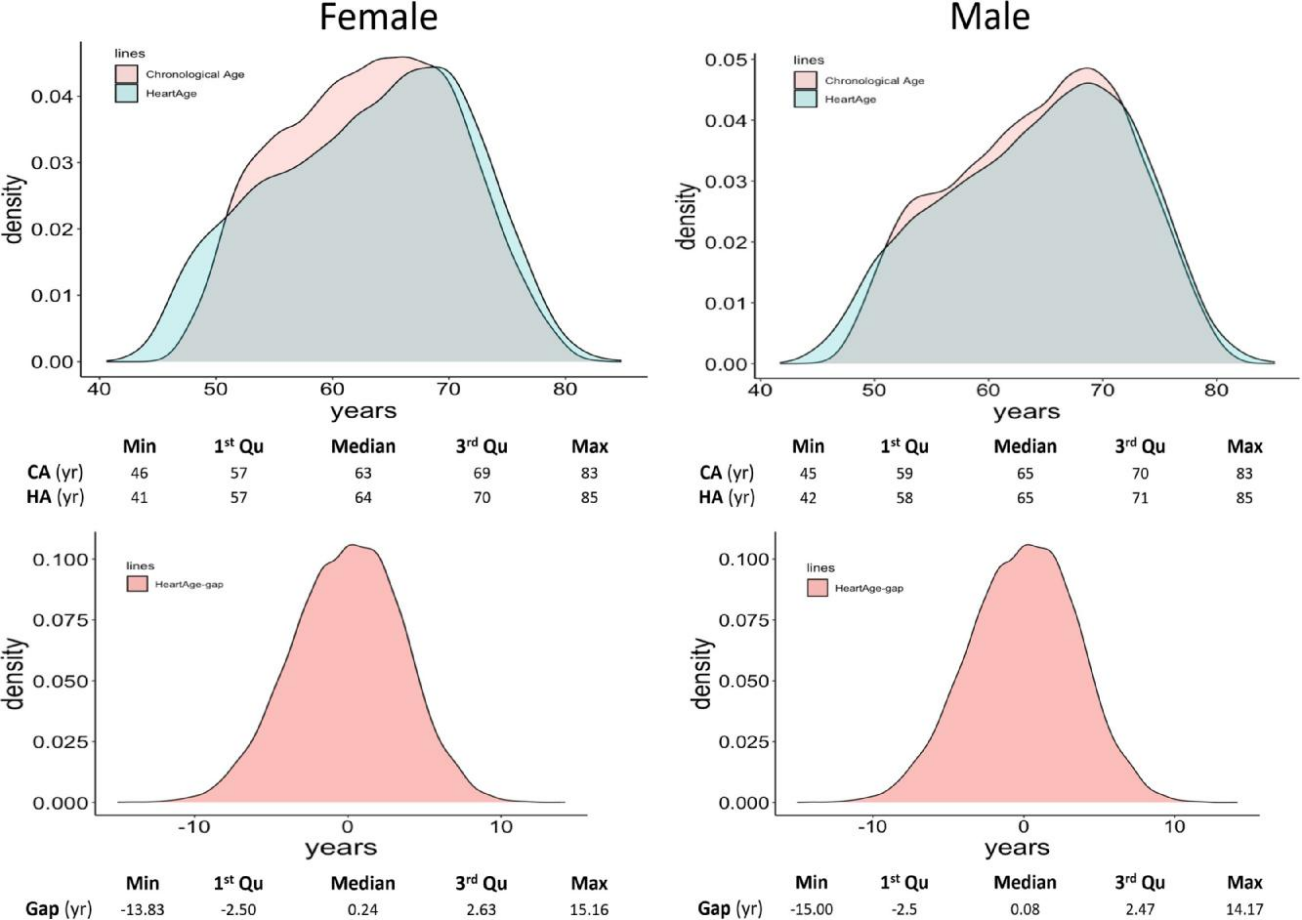
Distribution of chronological age, *HeartAge* and *HeartAge-gap*. Upper panel: density plots showing the distribution of chronological age and *HeartAge* (cardiovascular biological age estimate) in the UK-Biobank female and male participants. Bottom panel: density plots showing the distribution of *HeartAge-gap* (difference between biological age and chronological age) in the UK-Biobank male and female participants. Median, 1st-3rd quartiles (Qu), minimum (Min) and maximum (Max) values are shown at the bottom of each distribution. CA, Chronological age; HA, *HeartAge*; Gap, *HeartAge-gap*; yr, years.

In the study cohort, *HeartAge* exhibited a normal distribution that mirrored the distribution of chronological age in both males and females. Similarly, *HeartAge-gap*, which quantifies deviations from normative cardiovascular ageing at an individual level, displayed a normal distribution with a median value approximating 0, and extreme values of ∼±15 years in both sexes (*Figure 3*).

### *HeartAge-gap* and prevalent cardiovascular conditions

**Figure 4:**
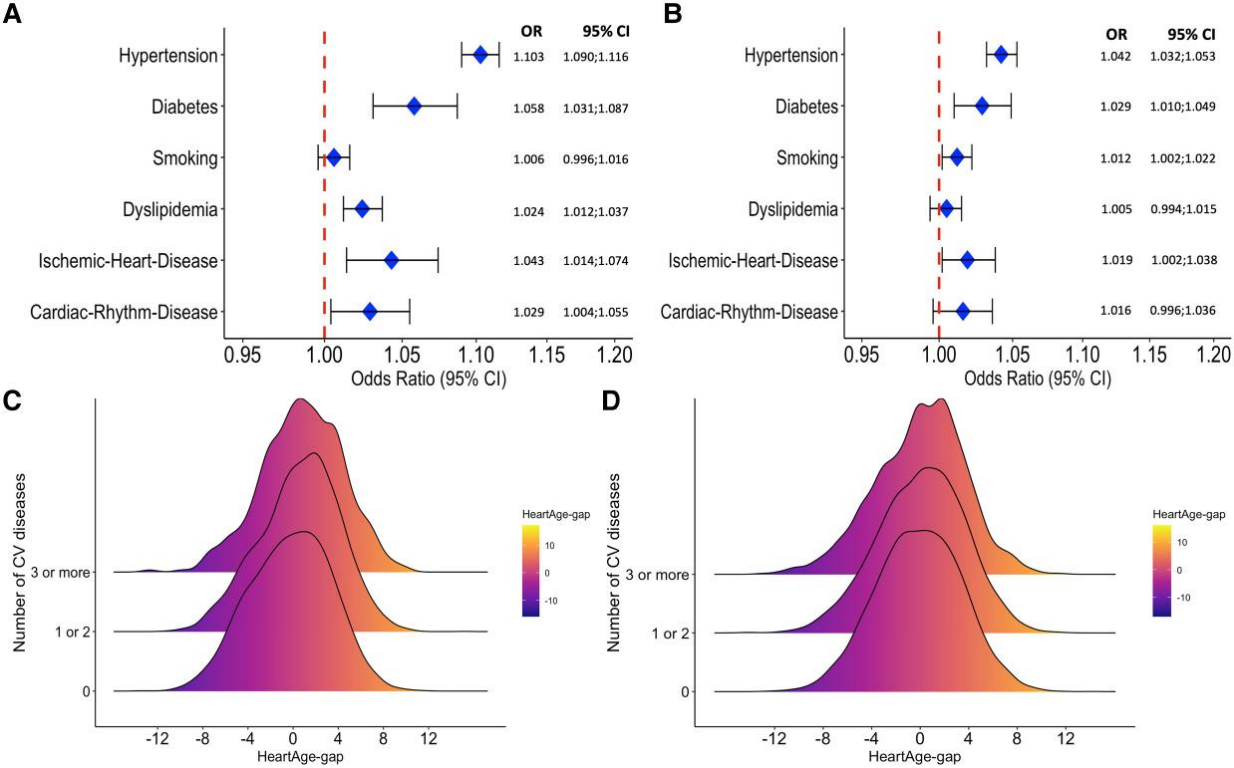
Association between *HeartAge-gap* and prevalent cardiovascular conditions. Upper panel: Forest plots of the odds ratios and 95% confidence intervals (CI) for the association between *HeartAge-gap* and prevalent cardiovascular (CV) conditions in female (*A*) and male (*B*) participants, respectively. All models were adjusted for chronological age. Lower panel: Distribution plots of *HeartAge-gap* in female (*C*) and male (*D*) participants according to the number of prevalent cardiovascular diseases. The distributions shift rightward, reflecting an older cardiovascular system, with increasing number of cardiovascular diseases.

Logistic regression models revealed that, in the study cohort, an increase in *HeartAge-gap* was associated with a higher likelihood of hypertension, diabetes, and ischaemic heart disease in both males and females (*Figure 4*). Additionally, in females, an increased *HeartAge-gap* was associated with cardiac rhythm abnormalities and dyslipidaemia. All models were adjusted for chronological age to assess how deviation from normative ageing, as captured by *HeartAge-gap,* associates with cardiovascular conditions independently of chronological age. Of note, the distribution of *HeartAge-gap* shifted rightward with increasing numbers of prevalent cardiovascular diseases in both males and females (*Figure 4*).

### *HeartAge-gap* and composite cardiovascular outcome

**Figure 5:**
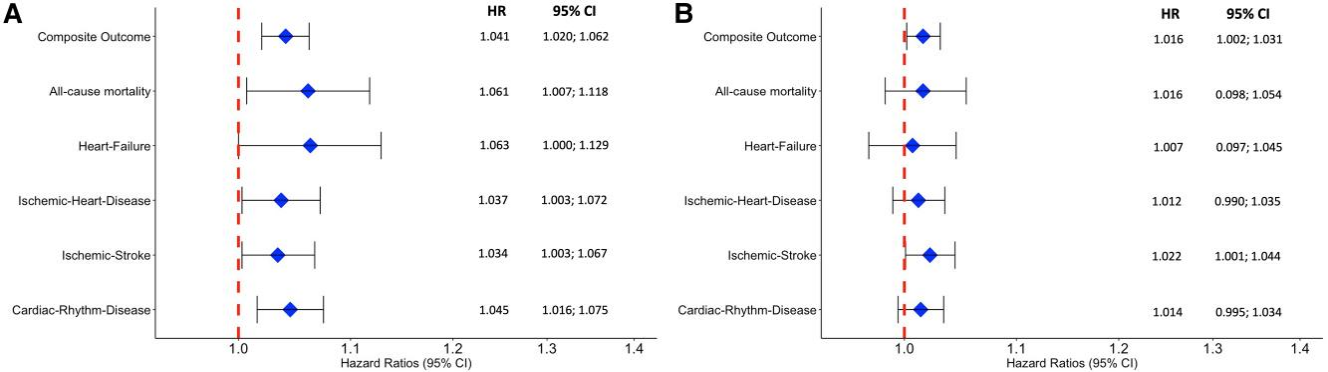
Association between *HeartAge-gap* and cardiovascular outcome. Forest plots showing the hazard ratios and 95% confidence intervals for the association between *HeartAge-gap*, the composite cardiovascular outcome, and its event breakdown in female (*A*) and male (*B*) participants, respectively. All models were adjusted for chronological age.

During a median follow-up of 5.9 years (interquartile-range: 4.7–7.2 years), 722 female participants experienced the composite cardiovascular outcome, yielding an annual event rate of 7.35 per 1000 females. Ten out of 16 640 female participants (0.04%) experienced cardiovascular death during follow-up. The parsimonious Cox regression proportional hazard-ratio model indicated that advanced cardiovascular ageing, as reflected by an increased *HeartAge-gap*, was associated with a higher likelihood of experiencing the composite cardiovascular endpoint, regardless of chronological age [HR:1.036 (95% CI:1.014–1.059), *P* = 0.001 per each year increment in *HeartAge-gap*]. Moreover, increased *HeartAge-gap* was associated with an increased odds of developing heart failure, ischaemic heart disease, ischaemic stroke, or cardiac rhythm abnormalities independently of chronological age (*Table 2*), (*Figure 5*). The fully-adjusted Cox regression proportional hazard-ratio model confirmed the association between an increased *HeartAge-gap* and the composite cardiovascular outcome after adjusting for chronological age and other major confounders at baseline [HR:1.022 (95% CI: 1.000–1.044), *P* = 0.048 per each year increment in *HeartAge-gap*], (*Table 2*, Supplementary data online, *Table S5*), (*Graphical Abstract*).

**Table 2:** Cox regression proportional hazard-ratio models for the composite cardiovascular outcome and its individual components

Outcome	Female			Male		
*Parsimonious Cox regression proportional hazard-ratio models*						
	HR	95% CI	*P*-value	HR	95% CI	*P*-value
**Composite Outcome**						
*HeartAge-gap*	1.036	1.014–1.059	0.001	1.019	1.004–1.035	0.015
Chronological age	1.087	1.075–1.099	<0.001	1.078	1.070–1.087	<0.001
**Cardiovascular mortality**						
*HeartAge-gap*	1.109	0.898–1.369	0.336	0.945	0.826–1.081	0.410
Chronological age	1.038	0.900–1.197	0.608	1.029	0.969–1.094	0.349
**Heart-Failure**						
*HeartAge-gap*	1.063	1.000–1.129	0.049	1.007	0.9702–1.045	0.720
Chronological age	1.129	1.094–1.166	<0.001	1.103	1.081–1.126	<0.001
**Ischaemic Heart Disease**						
*HeartAge-gap*	1.037	1.003–1.072	0.032	1.012	0.990–1.035	0.275
Chronological age	1.086	1.068–1.104	<0.001	1.060	1.048–1.072	<0.001
**Ischaemic Stroke**						
*HeartAge-gap*	1.034	1.003–1.067	0.033	1.022	1.001–1.044	0.041
Chronological age	1.091	1.074–1.109	<0.001	1.064	1.052–1.075	<0.001
**Cardiac-Rhythm Abnormalities**						
*HeartAge-gap*	1.045	1.016–1.075	0.002	1.014	0.995–1.034	0.159
Chronological age	1.088	1.073–1.104	<0.001	1.097	1.086–1.108	<0.001

*Fully adjusted Cox regression proportional hazard-ratio models*						
Composite Outcome	HR	95% CI	*P*-value	HR	95% CI	*P*-value
*HeartAge-gap*	1.022	1.000–1.044	0.048	1.017	1.002–1.033	0.0272
Chronological age	1.070	1.058–1.082	<0.001	1.066	1.057–1.075	<0.001
Body-mass-index	1.023	1.007–1.040	0.006	1.044	1.030–1.059	<0.001
Hypertension	1.855	1.576–2.183	<0.001	1.525	1.358–1.713	<0.001
Diabetes	1.380	1.029–1.851	0.032	1.205	1.012–1.434	0.036
Ischaemic heart disease	3.616	2.893–4.519	<0.001	2.319	2.017–2.667	<0.001

The fully-adjusted multivariable Cox regression models were adjusted for *HeartAge-gap*, chronological age, body-mass index, hypertension, diabetes, and prevalent ischaemic heart disease.

CI, Confidence interval; HR, Hazard Ratio.

In male participants, 1373 experienced the composite outcome during a median follow-up of 5.5 years (interquartile-range: 4.6–6.9 years), yielding an annual event rate of 16.5 per 1000 males. 23 out of 15 144 male participants (0.15%) experienced cardiovascular death during follow-up. The parsimonious Cox regression proportional hazard-ratio model revealed that an increased *HeartAge-gap* was associated with a higher likelihood of experiencing the composite cardiovascular outcome, regardless of chronological age [HR: 1.019 (95% CI: 1.004–1.035), *P* = 0.015 per each year increment in *HeartAge-gap*]. In the breakdown of the composite cardiovascular outcome, an increased *HeartAge-gap* was associated with ischaemic stroke after adjusting for chronological age [HR: 1.022 (95% CI: 1.001–1.044), *P* = 0.041] (*Table 2*), (*Figure 5*). In the fully-adjusted Cox regression proportional hazard-ratio model, *HeartAge-gap* was associated with the composite cardiovascular outcome after correction for chronological age and other major confounders at baseline [HR:1.017 (95% CI: 1.002–1.033), *P* = 0.027 per each year increment in *HeartAge-gap*], (*Table 2*, Supplementary data online, *Table S5*), (*Graphical Abstract*).

### *HeartAge-gap* and all-cause mortality

During follow-up, 123 female participants died resulting in an annual mortality rate of 1.25 per 1000 females. The Cox regression proportional hazard-ratio model indicated that advanced cardiovascular age, as reflected by an increased *HeartAge-gap*, was associated with higher hazard of all-cause mortality, after adjusting for chronological age [HR: 1.061 (95% CI: 1.007–1.118), *P* = 0.027 per each year increment in *HeartAge-gap*], (*Graphical Abstract*).

During the follow-up, 240 male participants died yielding an annular mortality rate of 2.88 per 1000 participants. No association was found between *HeartAge-gap* and all-cause mortality in the Cox regression proportional hazard-ratio model after adjusting for chronological age [HR:1.016 (95% CI: 0.9799–1.054), *P* = 0.382 per each year increment in *HeartAge-gap*], (*Graphical Abstract*).

### Association between *HeartAge-gap* and specific cardiovascular measures

The reduction of aortic distensibility with an increase in *HeartAge-gap* was more accentuated in males than females. Similarly, ventricular volumes and mass declined more rapidly with an increase in *HeartAge-gap* in males compared with females, with a similar trend being observed for atrial end-systolic volumes. In contrast, the ventricular systolic function increased more rapidly with an increase in *HeartAge-gap* in females than in males (*Table 3*).

**Table 3:** Associations of cardiovascular MRI phenotypes with HeartAge-gap stratified by sex

MRI Phenotypes	Female			Male		
	β−value	95% CIs	*P*-value	β−value	95% CIs	*P*-Value
AoD (10^−3^ mmHg^−1^)	−0.137	−0.140; −0.135	<0.001	−0.212	−0.124; −0.0119	<0.001
AoED (cm^2^)	0.169	0.162; 0.176	<0.001	0.156	0.147; 0.165	<0.001
AoES (cm^2^)	0.099	0.092; 0.107	<0.001	0.078	0.069; 0.087	<0.001
LV-EDV (mL)	−1.572	−1.660; −1.483	<0.001	−2.791	−2.934; −2.649	<0.001
LV-ESV (mL)	−0.725	−0.777; −0.673	<0.001	−1.154	−1.245; −1.063	<0.001
LV-mass (g)	−0.234	−0.283; −0.185	<0.001	−0.906	−0.986; −0.826	<0.001
LV-EF (%)	0.172	0.144; 0.199	<0.001	0.103	0.069; 0.137	<0.001
LA-conduit vol (mL)	−0.726	−0.754; −0.698	<0.001	−0.950	−0.988; −0.912	<0.001
LA-reservoir (mL)	−0.008	−0.057; 0.040	0.737	0.109	0.0349; 0.1036	0.004
LA-pump volume (mL)	0.187	0.157; 0.218	<0.001	0.072	0.0313; 0.113	<0.001
RV-EDV (mL)	−1.827	−1.935; −1.719	<0.001	−3.365	−3.529; −3.201	<0.001
RV-ESV (mL)	−1.010	−1.066; −0.954	<0.001	−1.698	−1.783; −1.613	<0.001
RV-EF (%)	0.186	0.157; 0.216	<0.001	0.139	0.107; 0.172	<0.001
RA-conduit volume (mL)	−0.078	−0.130; −0.026	0.003	−0.165	−0.255; −0.074	<0.001
RA-reservoir volume (mL)	−0.051	−0.132; 0.029	0.210	−0.114	−0.249; 0.021	0.099
RA-pump volume (mL)	0.277	0.244; 0.310	<0.001	0.283	0.232; 0.335	<0.001

β-values represent the linear association coefficients expressing change in each cardiovascular MRI phenotype per 1-year increment in *HeartAge-gap*.

Atrial volumes: LA volumes were derived from bi-planar analysis of 2− and 4-chamber cine images, and RA volumes from 4-chamber cine only. Volumes were sampled at: (i) ventricular end-systole (maximum atrial volume, reservoir), (ii) ventricular diastole immediately before atrial contraction (conduit), and (iii) ventricular end-diastole after atrial contraction (minimum atrial volume, pump). Accordingly, reservoir volume = maximal atrial volume, conduit volume = atrial volume pre-atrial contraction, and pump volume = minimal atrial volume.

Ascending Aortic Distensibility (AoD): The cross-sectional area of the ascending aorta was extracted across the cardiac cycle, with minimum (Amin) and maximum (Amax) areas identified. Distensibility was computed as (Amax—Amin)/(Amin × PP), where PP denotes central pulse pressure in mmHg measured at the time of MRI.

All models were adjusted for chronological age (yrs) and squared chronological age (yrs^2^), weight (kg) and height (cm).

AoD, ascending aortic distensibility; AoED, ascending aortic cross-sectional area at end-diastole; AoES, ascending aortic cross-sectional area at end-systole; CI, confidence interval; EDV, end-diastolic volume; EF, ejection fraction; ESV, end-systolic volume; LA, left atrium; LV, left ventricular; MRI, Magnetic Resonance Imaging; RA, right atrium; RV, right ventricular.

### Validation in the MESA cohort

The baseline characteristics and cardiovascular MRI measures of the 894 MESA participants are shown in the Supplementary data online, *Table S6*. The distribution of *HeartAge*(mesa) *and HeartAge-gap*(mesa) are shown in the Supplementary data online, *Figure S2*.

During a median follow-up of 17.8 years (interquartile-range: 13.5–18.5 years), 39 females participants experienced the composite cardiovascular outcome, yielding an annual event rate of 4.64 per 1000 females. Increased *HeartAge-gap*(mesa) was associated with an increased odds of developing the hard cardiovascular outcome [HR: 1.113 (95% CI: 1.025–1.210), *P* = 0.011 per each year increment in *HeartAge-gap*(mesa)] independently of chronological age and other major cofounders, including hypertension, diabetes and BMI at baseline. In males, during a median follow-up of 17.6 years (interquartile-range: 12.5–18.4 years), 57 participants developed the hard cardiovascular outcome, resulting in an annual event rate of 7.67 per 1000 males. No association was found between *HeartAge-gap*(mesa) and the composite cardiovascular outcome [HR: 1.026 (95% CI: 0.959–1.097), *P* = 0.458], (see Supplementary data online, *Table S7*).

## Discussion

Our approach enabled an estimate of cardiovascular biological age (*HeartAge*) and its deviation from chronological age (*HeartAge-gap*) using a supervised machine-learning model trained on easily extractable cardiovascular MRI phenotypes obtained from a standard clinical scan. Cross-sectional analysis revealed that an increased *HeartAge-gap* was consistently associated with a higher prevalence of hypertension, diabetes, and ischaemic heart disease in both sexes, regardless of chronological age. In females, each year increase in *HeartAge-gap* was associated with 10.3%, 5.8%, and 4.3% and increased odds of having hypertension, diabetes, or ischaemic heart disease, respectively. Importantly, over a median follow-up of nearly 6 years, *HeartAge-gap* was associated with the composite cardiovascular outcome independently of chronological age in both sexes, even after controlling for other key baseline confounders, including BMI, ischaemic heart disease, hypertension, and diabetes. In females, *HeartAge-gap* also emerged as a predictor of all-cause mortality regardless of chronological age, with each year increase in *HeartAge-gap* compounding a 6% increased risk of dying of any cause (*Graphical Abstract*).

To our knowledge, only two prior studies estimated the cardiovascular biological age using machine-learning models trained on cardiovascular MRI phenotypes.^13,16^ Raisi-Estabragh *et al*.^13^ employed Bayesian ridge regression, informed by MRI radiomics, which consists of a high-dimensional multi-feature array of cardiac shape and myocardial tissue features, to estimate cardiovascular biological age and the related age-gap, but lacked longitudinal validation against clinical outcome. Shah *et al*.^16^ utilized CatBoost gradient boosting incorporating 126 cardiovascular MRI phenotypes (including T1 mapping and frame-wise strain analysis) to estimate the cardiovascular biological age, but reported only a marginal association between quartiles of age-gap and cardiovascular outcomes, with no significant association when age-gap was introduced as a continuous variable in the Cox regression models. These limitations, coupled with the complexity in extracting and analysing large arrays of cardiovascular MRI phenotypes, limit the research and clinical transferability of these approaches. In contrast, our framework relies on a streamlined set of standard cardiovascular MRI phenotypes—ventricular and atrial volumes, function, and aortic distensibility—which can be easily extracted and quantified using either an automatic machine-learning segmentation pipeline, as applied in this current study, or semi-automatic post-processing method as routinely done in clinical practice. Furthermore, we externally validated our approach using the MESA cohort, demonstrating that *HeartAge-gap* predicted cardiovascular death, myocardial infarction or ischaemic stroke in female participants, regardless of chronological age and other major baseline confounders. The diverse ethnic composition and higher cardiovascular risk profile of the MESA cohort, compared with the predominantly low-risk European ancestry of the UK-Biobank, underscore the generalizability and potential clinical utility of our framework.

Interestingly, *HeartAge-gap* performed substantially better in females than in males. In the UK-Biobank cohort, females with an increased *HeartAge-gap* were more likely to experience the individual components of the composite cardiovascular outcome, including heart failure, ischaemic heart disease, cardiac rhythm abnormalities, or death from any cause. Similarly, in the MESA cohort, an increased *HeartAge-gap* was associated with the cardiovascular outcome only in female participants. Although an in-depth understanding of the age- and sex-specific complexity in ageing is beyond scope of this work, several factors may help to explain these results. First, the UK-Biobank cohort included a high proportion of elderly females (54%), which was also reflected in the model training. This sex imbalance may have helped fine-tune the model to better capture ageing patterns in females.^29^ Second, consistent with prior reports,^12,13,30,31^ we observed sex-specific differences in ageing. Compared with males, females showed a lower decline in aortic distensibility and cardiac chamber volumes, but a greater increase in biventricular systolic function with ageing. For instance, in females, left and right ventricular end-diastolic volumes decreased on average by 1.6% and 1.8% for each year increment in *HeartAge-gap*, respectively, as compared to 2.8% and 3.4% in males (*Table 3*). These differences may reflect underlying chromosomal, hormonal and biological factors that contribute to distinct ageing trajectories in males and females.^30,31^

Recently, Ladejobi *et al*.^32^ demonstrated that convolution-neural-network-enabled 12-lead ECG can predict biological age, with the ECG-derived age-gap associating with cardiovascular and all-cause mortality in more than 25 000 patients presenting to primary care. While our study and that of Ladejobi *et al*.^32^ share the overarching concept of biological age estimation, they are grounded in fundamentally different physiological domains. ECG-derived biological age captures electrical conduction and rhythm features, which may remain normal despite substantial structural or functional remodelling.^33^ By contrast, cardiovascular MRI-based *HeartAge* quantifies structural and functional parameters including ventricular volumes, myocardial mass, and aortic distensibility that integrate cumulative haemodynamic load and vascular ageing, thereby offering a direct window into cardiovascular morphology and function. In our study, the SHapley Additive exPlanations analysis, which measures the importance of each MRI phenotype in improving the machine-learning model,^25^ showed that aortic distensibility was the most important factor for *HeartAge* estimation. This finding was confirmed by linear regression models which disclosed an inverse relationship between advanced *HeartAge-gap* and aortic distensibility in both males and females. Aortic distensibility reflects the elastic properties of the proximal ascending aorta, and its decline with ageing indicates the replacement of elastin with collagen fibres in the vessel wall leading to vascular stiffening, adverse ventricular remodelling and dysfunction, and increased cardiovascular mortality and morbidity.^34,35^ Therefore, lifestyle or drug-based strategies aimed at mitigating the decline of aortic distensibility could play an instrumental role in tackling the accelerated ageing of the cardiovascular system, with potentially far-reaching consequences on cardiovascular health.^35^

Our novel cardiovascular ageing framework holds potential for both cardiovascular research and prevention. In research, unbiased data-driven approaches could leverage *HeartAge-gap* to identify novel molecular signatures of cardiovascular ageing by exploring its associations with multi-omics data, opening new avenues for *in-vitro* and *in-vivo* mechanistic experimental studies for novel druggable ageing pathways. Additionally, *HeartAge-gap* could serve as a surrogate endpoint in randomized controlled trials evaluating repurposed anti-ageing therapy in humans, circumventing the need for lengthy and costly trials testing treatments against morbidity and mortality.^4,5^ In preventive medicine, our framework offers the potential to empower personalized strategies to alleviate cardiovascular disease burden. Measuring *HeartAge-gap* in early adulthood, when biological age starts diverging from chronological age, could enable timely lifestyle modifications or drug-based interventions to postpone cardiovascular diseases onset and progression.^4,5^ The implementation of this approach, particularly when benchmarked against the high societal and economic costs of chronic cardiovascular diseases at the population level,^1,36^ is within reach due to the rapid advancements in low-cost low-field MRI technology,^37^ and the exponential growth in computational power, which supports the deployment of high-performance machine-learning algorithms.^38^ Rather, the immediate translational opportunity lies in capitalizing on clinically indicated cardiovascular MRI scans in which *HeartAge-gap* can be seamlessly integrated into existing post-processing workflows or estimated inline at the scanner, thereby providing additional prognostic information as a by-product of the standard report, without extending scan time or incurring extra cost.

### Limitations

Firstly, we cross-sectionally estimated cardiovascular biological age, therefore, *HeartAge-gap* reflected between-individual ageing trajectory, not allowing within-person assessment. However, prior studies reported that, although longitudinal measures are desirable, cross-sectional studies can reliably estimate biological age by capturing factors that are not fully related to chronological age, but nonetheless important in ageing.^39,40^ The absence of sex- and age-specific cardiovascular MRI reference thresholds for LVEF may have introduced some degree of misclassification, although our chosen >50% cut-off is supported by large-scale CMR reference data in adults over 40 years.^23^ Although stringent clinical and imaging criteria were applied to exclude individuals with overt cardiovascular diseases or risk factors, we cannot fully account for latent genetic predispositions of myocardial or vascular function, which may not be detectable through conventional clinical and imaging data. While we applied a comprehensive case-finding strategy by cross-referencing self-reported disease and risk factors (UK-Biobank data-field 3001), hospital episode statistics (2000), cancer registry (100 092), and first-occurrence records (1712), we acknowledge that some individuals may have had undiagnosed hypertension, diabetes, or dyslipidaemia at baseline. Such unrecognized disease could not be identified from available data and may have led to inadvertent inclusion within the healthy reference cohort. The UK-Biobank cohort comprised of individuals that were mainly elderly, predominantly female (54%), of European ancestry, and residing in relatively affluent regions across the UK. These factors therefore limit the generalizability of our results. Moreover, this was a retrospective, observational study and, as such, causality cannot be inferred. Several clinically relevant variables such as renal function, chronic lung disease, and anaemia were not available and therefore could not be incorporated into the multivariable models. These unmeasured factors may have influenced the outputs of the logistic and Cox regression models and should be considered when interpreting the findings of this study. Furthermore, *HeartAge-gap* was not associated with cardiovascular death in either sex. This was likely due to the small number of events (e.g. only ten cardiovascular deaths amongst UK-Biobank females). Left ventricular diastolic function, an important marker of cardiovascular ageing, was not incorporated into the *HeartAge* (XGBoost) model, and this limitation should be considered when interpreting our findings. Finally, MESA participants were followed-up for a substantially longer interval time as compared to the UK-Biobank participants. Given that the impact of cardiovascular risk factors on clinical outcome declines as the follow-up increases,^41^ the association of *HeartAge-gap* with the cardiovascular outcome in the MESA study was likely diluted.

## Conclusion

We developed and validated a framework for estimating cardiovascular biological ageing (*HeartAge*) using a XGBoost model informed by routinely measurable cardiovascular MRI phenotypes. The deviation between biological and chronological age (*HeartAge-gap*) emerged as an independent, albeit modest, correlate of cardiovascular outcomes in both sexes and, in females, of all-cause mortality.

## Supplementary Material

jeaf337_Supplementary_Data

## Data Availability

The data underpinning this study will be shared on reasonable request by the corresponding author.
