# Correction to: A streamlined CMR-derived machine-learning model for estimating cardiovascular biological age: development and validation in the UK-biobank and multi-ethnic study of atherosclerosis

**DOI:** 10.1093/ehjci/jeag175

**Published:** 2026-07-14

This is a correction to: Pier-Giorgio Masci, Gianni Andreozzi, Esther Puyol-Anton, Ashkan Abdollahi, Richard Mospan, Bram Ruijsink, Marina Cecelja, Phillip J Chowienczyk, Aqeel T Mohamed, Alistair Young, Bharath Ambale, Amedeo Chiribiri, Geoff Tison, Claire J Steves, Joao A C Lima, Reza Razavi, Valentina Lorenzoni, Andrew King, A streamlined CMR-derived machine-learning model for estimating cardiovascular biological age: development and validation in the UK-biobank and multi-ethnic study of atherosclerosis, *European Heart Journal - Cardiovascular Imaging*, Volume 27, Issue 3, March 2026, Pages 515–526, https://doi.org/10.1093/ehjci/jeaf337

In the originally published version of this manuscript, Table 1 contained errors under the ‘MRI Phenotypes’ heading. In addition, some text below the table was inadvertently omitted.

Table 1 has been corrected to read:

**Table 1.** Baseline characteristics of the study cohort

	Female (*n* = 16 640)	Male (*n* = 15 144)
**Baseline characteristics**		
Age (years)	63.12 ± 7.36	64.45 ± 7.63
Body-mass-index (kg/m^2^)	25.82 ± 4.48	26.89 ± 3.86
Systolic blood pressure (mmHg)	136.89 ± 20.16	143.14 ± 18.29
Diastolic blood pressure (mmHg)	76.76 ± 10.45	80.51 ± 10.34
Heart rate (bpm)	70.43 ± 11.18	67.01 ± 12.19
Active smoking, *n* (%)	4831 (33.4%)	5388 (40.68%)
Diabetes, *n* (%)	485 (2.9%)	941 (6.2%)
Hypertension, n (%)	3285 (19.7%)	4618 (30.49%)
Dyslipidemia, *n* (%)	2649 (15.9%)	4003 (26.4%)
Ischaemic heart disease, *n* (%)	437 (2.6%)	1102 (7.2%)
Cardiac rhythm abnormalities, *n* (%)	557 (3.3%)	883 (5.8%)
**MRI Phenotypes**		
LV end-diastolic volume (mL)	131.99 ± 24.41	170.88 ± 34.66
LV end-systolic volume (mL)	43.11 ± 12.96	60.69 ± 20.07
LV stroke volume (mL)	88.88 ± 16.65	110.2 ± 22.44
LV ejection fraction (%)	67.59 ± 6.21	64.85 ± 7.01
LV end-diastolic mass (g)	78.63 ± 13.66	111.8 ± 20.02
RV end-diastolic volume (mL)	141.99 ± 28.45	188.31 ± 40.64
RV end-systolic volume (mL)	55.29 ± 14.26	78.02 ± 20.53
RV stroke volume (%)	86.7 ± 19.93	110.29 ± 27.35
RV ejection fraction (%)	60.99 ± 6.68	58.47 ± 6.77
RA reservoir volume (mL)	71.31 ± 18.87	96.74 ± 29.3
RA conduit volume (mL)	35.17 ± 12.09	50.39 ± 19.49
RA pump volume (mL)	21.19 ± 7.69	27.81 ± 11.01
LA reservoir volume (mL)	64.81 ± 18.34	75.54 ± 24.53
LA conduit volume (mL)	21.96 ± 10.96	27.09 ± 15.72
LA pump volume (mL)	20.1 ± 7.09	24.43 ± 8.76
Ascending aorta area at end-diastole (cm^2^)	7.93 ± 1.8	9.36 ± 2.08
Ascending aorta area at end-systole (cm^2^)	8.62 ± 1.74	10.19 ± 2.06
Ascending aorta distensibility (10^−3^ mmHg^−1^)	0.002 ± 0.001	0.002 ± 0.001
Basal anterior wall thickness (mm)	7.02 ± 1.02	8.2 ± 1.24
Basal anterior septum thickness (mm)	6.29 ± 1.39	7.5 ± 1.62
Basal inferior septum thickness (mm)	5.56 ± 1.19	6.61 ± 1.44
Basal inferior wall thickness (mm)	6.09 ± 0.81	7.07 ± 0.99
Basal inferolateral wall thickness (mm)	5.81 ± 0.75	6.73 ± 0.96
Basal anterolateral wall thickness (mm)	6.12 ± 0.77	7.11 ± 1.01
Mid anterior wall thickness (mm)	5.39 ± 0.57	6.27 ± 0.8
Mid anterior septum thickness (mm)	6.42 ± 0.8	7.7 ± 1
Mid inferior septum thickness (mm)	6.63 ± 0.94	8.18 ± 1.12
Mid inferior wall thickness (mm)	5.79 ± 0.73	6.89 ± 0.92
Mid inferolateral wall thickness (mm)	5.28 ± 0.63	6.24 ± 0.88
Mid anterolateral wall thickness (mm)	5.31 ± 0.52	6.21 ± 0.81
Apical anterior wall thickness (mm)	5.23 ± 0.62	5.74 ± 0.66
Apical septal thickness (mm)	5.47 ± 0.79	6.47 ± 0.86
Apical inferior wall thickness (mm)	4.6 ± 0.74	5.5 ± 0.81
Apical lateral wall thickness (mm)	4.93 ± 0.67	5.6 ± 0.75
Average LV wall thickness (mm)	5.81 ± 0.55	6.81 ± 0.7

LA: Left atrium; LV: Left ventricular; MRI: Magnetic Resonance Imaging; RA: Right atrium; RV: Right ventricular

Atrial volumes: LA volumes were derived from bi-planar analysis of 2- and 4-chamber cine images, and RA volumes from 4-chamber cine only. Volumes were sampled at: (1) ventricular end-systole (maximum atrial volume, reservoir), (2) ventricular diastole immediately before atrial contraction (conduit), and (3) ventricular end-diastole after atrial contraction (minimum atrial volume, pump). Accordingly, reservoir volume = maximal atrial volume, conduit volume = atrial volume pre-atrial contraction, and pump volume = minimal atrial volume.

Ascending Aortic Distensibility (AoD): The cross-sectional area of the ascending aorta was extracted across the cardiac cycle, with minimum (Amin) and maximum (Amax) areas identified. Distensibility was computed as (Amax − Amin) / (Amin × PP), where PP denotes central pulse pressure in mmHg measured at the time of MRI.

Instead of:

**Table 1:** Baseline characteristics of the study cohort

	Female (*n* = 16 640)	Male (*n* = 15 144)
**Baseline characteristics**		
Age (years)	63.12 ± 7.36	64.45 ± 7.63
Body-mass-index (kg/m^2^)	25.82 ± 4.48	26.89 ± 3.86
Systolic blood pressure (mmHg)	136.89 ± 20.16	143.14 ± 18.29
Diastolic blood pressure (mmHg)	76.76 ± 10.45	80.51 ± 10.34
Heart rate (bpm)	70.43 ± 11.18	67.01 ± 12.19
Active smoking, *n* (%)	4831 (33.4%)	5388 (40.68%)
Diabetes, *n* (%)	485 (2.9%)	941 (6.2%)
Hypertension, *n* (%)	3285 (19.7%)	4618 (30.49%)
Dyslipidemia, *n* (%)	2649 (15.9%)	4003 (26.4%)
Ischaemic heart disease, *n* (%)	437 (2.6%)	1102 (7.2%)
Cardiac rhythm abnormalities, *n* (%)	557 (3.3%)	883 (5.8%)
**MRI Phenotypes**		
LV end-diastolic volume (mL)	131.99 ± 24.41	170.88 ± 34.66
LV end-systolic volume (mL)	43.11 ± 12.96	60.69 ± 20.07
LV stroke volume (mL)	88.88 ± 16.65	110.2 ± 22.44
LV ejection fraction (%)	67.59 ± 6.21	64.85 ± 7.01
LV end-diastolic mass (g)	78.63 ± 13.66	111.8 ± 20.02
RV end-diastolic volume (mL)	141.99 ± 28.45	188.31 ± 40.64
RV end-systolic volume (mL)	55.29 ± 14.26	78.02 ± 20.53
RV stroke volume (%)	86.7 ± 19.93	110.29 ± 27.35
RV ejection fraction (%)	60.99 ± 6.68	58.47 ± 6.77
RA end-diastolic volume (mL)	35.17 ± 12.09	50.39 ± 19.5
RA end-systolic volume (mL)	71.31 ± 18.87	96.74 ± 29.3
RA reservoir volume (mL)	14.01 ± 6.84	17.25 ± 9.38
RA pump volume (mL)	21.19 ± 7.69	27.82 ± 11.01
LA end-diastolic volume (mL)	21.96 ± 10.96	27.1 ± 15.72
LA end-systolic volume (mL)	64.81 ± 18.34	75.54 ± 24.53
LA reservoir volume (mL)	21.31 ± 7.52	22.84 ± 9.18
LA pump volume (mL)	20.1 ± 7.09	24.43 ± 8.76
Ascending aorta area at end-diastole (cm^2^)	7.93 ± 1.8	9.36 ± 2.08
Ascending aorta area at end-systole (cm^2^)	8.62 ± 1.74	10.19 ± 2.06
Ascending aorta distensibility (10^−3^ mmHg^−1^)	0.002 ± 0.001	0.002 ± 0.001
Basal anterior wall thickness (mm)	7.02 ± 1.02	8.2 ± 1.24
Basal anterior septum thickness (mm)	6.29 ± 1.39	7.5 ± 1.62
Basal inferior septum thickness (mm)	5.56 ± 1.19	6.61 ± 1.44
Basal inferior wall thickness (mm)	6.09 ± 0.81	7.07 ± 0.99
Basal inferolateral wall thickness (mm)	5.81 ± 0.75	6.73 ± 0.96
Basal anterolateral wall thickness (mm)	6.12 ± 0.77	7.11 ± 1.01
Mid anterior wall thickness (mm)	5.39 ± 0.57	6.27 ± 0.8
Mid anterior septum thickness (mm)	6.42 ± 0.8	7.7 ± 1
Mid inferior septum thickness (mm)	6.63 ± 0.94	8.18 ± 1.12
Mid inferior wall thickness (mm)	5.79 ± 0.73	6.89 ± 0.92
Mid inferolateral wall thickness (mm)	5.28 ± 0.63	6.24 ± 0.88
Mid anterolateral wall thickness (mm)	5.31 ± 0.52	6.21 ± 0.81
Apical anterior wall thickness (mm)	5.23 ± 0.62	5.74 ± 0.66
Apical septal thickness (mm)	5.47 ± 0.79	6.47 ± 0.86
Apical inferior wall thickness (mm)	4.6 ± 0.74	5.5 ± 0.81
Apical lateral wall thickness (mm)	4.93 ± 0.67	5.6 ± 0.75
Average LV wall thickness (mm)	5.81 ± 0.55	6.81 ± 0.7

LA, Left atrium; LV, Left ventricular; MRI, Magnetic Resonance Imaging; RA, Right atrium; RV, Right ventricular.

