# Supplementary material for: A streamlined CMR-derived machine-learning model for estimating cardiovascular biological age: development and validation in the UK-biobank and multi-ethnic study of atherosclerosis
Source: Eur Heart J Cardiovasc Imaging. 2025 Dec 4;27(3):515–26. doi: 10.1093/ehjci/jeaf337 (PMC13367178; doi:10.1093/ehjci/jeaf337)
Supplement: jeaf337_Supplementary_Data [file jeaf337_supplementary_data.pdf]

# Supplemental Material

# Supplemental Methods

### Development and hyper-parametrisation of the XGBoost model in the UK-Biobank

For model development, fine tuning (hyper-parametrisation), and cross-validation we used the following R library (“xgboost”, “caret”).

The following hyper-parameter values were used to optimise the model in the training set (n=3,008):

- nrounds = c(500, 1000, 2000, 3000) [number of trees]
- max_depth = c(2,4,6) [maximum depth of a tree]
- eta = c(0.025, 0.05, 0.1, 0.3) [lelarning rate: scale the contribution of each tree by a factor of 0 < eta < 1 ]
- gamma = c(0, 0.05, 0.1, 0.5, 0.7, 1.0) [minimum loss reduction required to make a further partition on a leaf node of the tree]
- colsample_bytree = c(0.025, 0.50, 0.75, 1),
- min_child_weight = c(1,2,3) [minimum sum of instance weight (hessian) needed in a child]
- subsample = c(0.5, 0.75, 1.0) [subsample ratio of the training instance]

A five-fold cross-validation approach was used for hyperparameter optimisation. Based upon the cross-validation results, the best XGBoost model was identified by the lowest mean squared error (RMSE) in the prediction of chronological age.

```
train_control <- trainControl(method = "cv",
                              number=5,
                              verboseIter = TRUE,
                              returnData = FALSE,
                              returnResamp = "all",
                              classProbs = TRUE,
                              allowParallel = TRUE)

set.seed (1)
xgb_tune <- train (x = train_matrix_new,
                   y = train_ca_new,
                   trControl= train_control,
                   tuneGrid = grid_tune,
                   method = "xgbTree",
                   verbose = TRUE)
```

The hyperparameter optimisation process yielded the following values: nrounds = 500, eta=0.025, max_depth =2, colsample_bytree =1, min_child_weight =2, subsample = 0.5. The model was then trained with the hyperparameter values in the training set and tested using the ‘predict’ function in the held-out sample (test-set).

### Deployment of the XGBoost model in the Multi-Ethnic Study of Atherosclerosis cohort

We first hyper-parametrised the model in the training set of the UK-Biobank (n=3,008) using the same grid of hyper-parameters. However, we excluded the cardiovascular magnetic

resonance imaging (MRI) phenotypes that were not present in the Multi-Ethnic Study of Atherosclerosis (MESA) cohort, namely the 16-AHA wall thickness and average LV wall thickness (n=18 variables), (**Supplemental Table-3**). The hyperparameter optimisation process yielded the following values: nrounds = 500, eta=0.025, max_depth =2, colsample_bytree =0.75, min_child_weight =2, subsample = 0.5. We then applied the XGBoost model to the 894 participants of the MESA cohort.

The full R code is provided in the following GitHub repositor:
https://github.com/GianniAndreozzi/HeartAge

# Supplemental Tables

## Table-1. Diagnostic codes used to identify prevalent cardiovascular risk factors and diseases, and to select healthy individuals.

Prevalent cardiovascular risk factors and diseases were identified through ICD-9, ICD-10 and specific UK-Biobank categories / data-field antecedent to the date of cardiovascular magnetic resonance imaging (UK-Biobank data-field 53, instance 2).

| | **Self-reported** | **Hospital episode statistics** | **First occurrence** |
|---|---|---|---|
| **Hypertension** | Hypertension [1065], Essential hypertension [1072], Gestational hypertension/pre-eclampsia [1073] | Essential (primary) hypertension [I10, 401], Hypertensive heart disease [I11, I11.0, I11.9, 402], Hypertensive chronic kidney disease [I12, I12.0, I12.9, 403], Hypertensive heart and chronic kidney disease [I13, I13.0, I13.1, I13.2, 404], Secondary Hypertension [I15, I15.0, I15.1, I15.2, I15.8, I15.9, 405] | Essential (primary) hypertension [131287], Hypertensive heart disease [131289], Hypertensive renal disease [131291], Hypertensive heart and renal disease [131292], Secondary hypertension [131295] |
| **Diabetes** | Diabetes [1220], Type 1 diabetes [1222], Type 2 diabetes [1223] | | Non-insulin dependent diabetes mellitus [130709], Other specified diabetes mellitus [130713], Unspecified diabetes mellitus [130715], Insulin-dependent diabetes mellitus [130707] |
| **Smoking** | Smoking status 20116, never, previous, current | | |
| **Dyslipidemia** | Disorders of lipoprotein metabolism [E78] | | |
| **Long standing illness disability or infirmity** | 2188 | | |

| | | | |
|---|---|---|---|
| **Ischemic Heart Disease** | Angina [1074], Heart attack/myocardial infarction [1075] | Acute myocardial infarction [I21, I21.0, I21.1, I21.2, I21.3, I21.4, I21.9, 410], Subsequent ST elevation and non ST elevation myocardial infarction [I22, I22.0, I22.1, I22.2, I22.8, I22.9], Complications following STEMI and NSTEMI myocardial infarction [I23, I23.0, I23.1, I23.2, I23.3, I23.4, I23.5, I23.6, I23.8], Dressler's syndrome [I24.1], Old myocardial infarction [I25.2, 412], Angina pectoris [413] | Angina pectoris [131297], Acute myocardial infarction [131299], Subsequent myocardial infarction [131301], Complications following acute myocardial infarction [131303], Other acute ischaemic heart diseases [131305], Chronic ischaemic heart disease [131307] |
| **Ischemic stroke** | | Cerebral infarction [I63, I63.0, I63.1, I63.2, I63.3, I63.4, I63.5, I63.6, I63.8, I63.9], Stroke, not specified as haemorrhage or infarction [I64], Occlusion of cerebral arteries [434.0, 434.1, 434.9, 436] | |
| **Subarachnoid haemorrhage** | | Subarachnoid haemorrhage[I60.0, I60.1, I60.2, I60.3, I60.4, I60.5, I60.6, I60.7, I60.8, I60.9, 430] | |
| **Cerebral haemorrhage** | | Intracerebral haemorrhage [I61.0, I61.1, I61.2, I61.3, I61.4, I61.5, I61.6, I61.8, I61.9, 431] | |
| **Cardiac rhythm abnormalities** | Heart arrhythmia [1077], Atrial flutter [1483] | Atrioventricular and left bundle-branch block [I44], Other conduction disorder [I45, I45.0, I45.1, I45.2, I45.3, I45.4, I45.5, 426], Atrial fibrillation and flutter [I48, 427.3], | Atrioventricular and left bundle-branch block [131343], Other conduction disorders [131345], Atrial fibrillation and flutter [131351], Other cardiac arrhythmias [131353], Cardiac arrest [131347] |
| **Vascular disease** | Leg claudication/ intermittent claudication [1087], Arterial embolism [1088], Pulmonary embolism +/- dvt [1093], Vasculitis [1372], | | Subarachnoid haemorrhage [131361], Intracerebral haemorrhage [131363], Cerebral infarction [131367], Stroke, not specified as haemorrhage or infarction [131369], |

| | | | |
|---|---|---|---|
| | Microscopic polyarteritis [1379], Polyarteritis nodosa [1380], Aortic aneurysm rupture [1591], Aortic dissection [1592] | | Atherosclerosis [131386], Other peripheral vascular diseases [131387], Arterial embolism and thrombosis [131389], Occlusion and stenosis of precerebral arteries, not resulting in cerebral infarction [131371], Occlusion and stenosis of cerebral arteries, not resulting in cerebral infarction [131373], Transient cerebral ischaemic attacks and related syndromes [131057], Aortic aneurysm and dissection [131383], Other aneurysm [131385] |
| **Heart Failure** | Heart failure/pulmonary odema [1076], Cardiomyopathy[1079], Myocarditis [1426], Hypertrophic cardiomyopathy (HCM / HOCM) [1588] | Heart failure [I50, I50.1, I50.9, 428] | Heart failure [131355], Pulmonary oedema [131525], Cardiomyopathy [131339], Cardiomyopathy in diseases classified elsewhere [131341], Acute myocarditis [131335] |
| **Metabolic disease** | Hyperaldosteronism/Conn's syndrome [1235], Phaeochromocytoma [1236] | | Disorder of lipoprotein metabolism and other lipidaemias [130815] |
| **Valvular heart disease** | Rheumatic fever [1479], Mitral valve prolapse [1488], Mitral stenosis [1489], Aortic stenosis | | Rheumatic mitral valve diseases [131277], Rheumatic aortic valve disease [131280], Rheumatic tricuspid valve diseases [131281], Multiple valve diseases [131283], |

| | | | |
|---|---|---|---|
| | [1490], Aortic aneurysm [1492] | | Nonrheumatic mitral valve disorders [131323], Nonrheumatic aortic valve disorders [131325], Nonrheumatic tricuspid valve disorders [131327], Pulmonary valve disorders [131328], Endocarditis, valve unspecified [131331], Endocarditis and heart valve disorders in diseases classified elsewhere [131333] |
| **Brain/Neurodegenerative disease** | | Dementia in Alzheimer disease [F00.0, F00.1, F00.2, F00.9], Vascular dementia [F01.0, F01.1, F01.2, F01.3, F01.8, F01.9], Dementia in other diseases classified elsewhere [F02.0, F02.1, F0.2.2, F02.3, F02.4, F02.8], Unspecified dementia [F03], Alzheimer disease [G30.0, G30.1, G30.8, G30.9, 331.0] Other degenerative diseases of nervous system, not elsewhere classified [G31.0, G31.1, G31.2, G31.8], Parkinson disease [G20, 332.0], Secondary parkinsonism [G21.0, G21.1, G21.2, G21.3, G21.4, G21.8, G21.9, 332.1], Parkinsonism in diseases, classified elsewhere [G22], Other degenerative diseases of basal ganglia [G23.0, G23.1, G23.2, G23.3, G23.8, G23.9, 333.0]<br><br>Senile dementia with delusional or depressive features [290.2], Senile dementia with delirium [290.3], Alcohol-induced persisting dementia [291.2], Dementia in conditions classified elsewhere [294.1], Senile degeneration of brain [331.2] | Dementia in Alzheimer’s disease [130837], Vascular dementia [130839], Dementia in other diseases classified elsewhere [130841], Unspecified dementia [130843], Parkinson’s disease [131023], Secondary parkinsonism [131025], Alzheimer’s disease [131037], Other degenerative diseases of nervous system [131039], Multiple sclerosis [131043], Hemiplegia [131103], Paraplegia and tetraplegia [131105], Other paralytic syndromes [131107] |
| **Lung disease** | Asthma [1111], Chronic obstructive airways disease/COPD [1112], | Emphysema [J43, J43.0, J43.1, J43.2, J43.8, J43.9], Other chronic obstructive pulmonary disease [J44.0, J44.1, J44.8, J44.9] | Emphysema [131490], Other chronic obstructive pulmonary disease [131493], Asthma |

| | | | |
|---|---|---|---|
| | Emphysema/chronic bronchitis [1113], Bronchiectasis [1114], Interstitial respiratory [1115], Asbestosis [1120], Pulmonary fibrosis [1121], fibrosing Alveolitis/unspecified alveolitis [1122], Respiratory failure [1124], Sarcoidosis [1371] | | [131495], Status asthmaticus [131497], Bronchiectasis [131499], Respiratory failure, not elsewhere classified [131547], Other interstitial pulmonary diseases [131529], Pulmonary embolism [131309], Other pulmonary heart diseases [131311], Other diseases of pulmonary vessels [131313], Sarcoidosis [130687] |
| **Liver disease** | Liver failure/cirrhosis [1158], Alcoholic liver / alcoholic cirrhosis [1604] | Hepatic failure, not elsewhere classified [K72, K72.0, K72.1, K72.9], Chronic hepatitis, not elsewhere classified [K73.0, K73.1, K73.2, K73.8, K73.9], Fibrosis and cirrhosis of liver [K74.0, K74.1, K74.2, K74.3, K74.4, K74.5, K74.6], Cholelithiasis [K80.0, K80.1, K80.2, K80.3, K80.4, K80.5, K80.8] Cholecystitis [K81.0, K81.1, K81.8, K81.9] | Hepatic failure, not elsewhere classified [131529], Chronic hepatitis, not elsewhere classified [131663], Fibrosis and cirrhosis of liver [131667], Alcoholic liver disease [131659] |
| **Cancer** | Carcinoid syndrome/tumour [1432] | Malignant neoplasm of lip [C00, C00.0, C00.1, C00.2, C00.3, C00.4, C00.5, C00.6, C00.8, C00.9, 140], Malignant neoplasm of base of tongue [C01], Malignant neoplasm of other and unspecified parts of tongue [C02, C02.0, C02.1, C02.2, C02.3, C02.4, C02.8, C02.9, 141], Malignant neoplasm of gum [C03, C03.0, C03.1, C03.9, 143], Malignant neoplasm of floor of mouth [C04, C04.0, C04.1, C04.8, C04.9, 144], Malignant neoplasm of palate [C05, C05.0, C05.1, C05.2, C05.8, C05.9], Malignant neoplasm of other and unspecified parts of mouth [C06, C06.0, C06.1, C06.2, C06.8, C06.9, 145], Malignant neoplasm of parotid gland [C07], Malignant neoplasm of other and unspecified major salivary glands [C08, C08.0, C08.1, C08.8, 142], Malignant neoplasm of tonsil [C09, C09.0, C09.1, C09.8, C09.9], Malignant neoplasm of oropharynx [C10, C10.0, | |

| | | | |
|---|---|---|---|
| | | C10.1, C10.2, C10.3, C10.4, C10.8, C10.9, 146], Malignant neoplasm of nasopharynx [C11, C11.0, C11.1, C11.2, C11.3, C11.8, C11.9, 147], Malignant neoplasm of pyriform sinus [C12], Malignant neoplasm of hypopharynx [C13, C13.0, C13.1, C13.2, C13.8, C13.9, 148], Malignant neoplasm of other and ill-defined sites in the lip, oral cavity and pharynx [C14, C14.0, C14.2, C14.8, 149], Malignant neoplasm of esophagus [C15, C15.0, C15.1, C15.2, C15.3, C15.4, C15.5, C15.8, C15.9, 150], Malignant neoplasm of stomach [C16, C16.0, C16.1, C16.2, C16.3, C16.4, C16.5, C16.6, C16.8, C16.9, 151], Malignant neoplasm of small intestine [C17, C17.0, C17.1, C17.2, C17.3, C17.8, C17.9, 152], Malignant neoplasm of colon [C18, C18.0, C18.1, C18.2, C18.3, C18.4, C18.5, C18.6, C18.7, C18.8, C18.9, 152], Malignant neoplasm of rectosigmoid junction [C19, 154], Malignant neoplasm of rectum [C20], Malignant neoplasm of anus and anal canal [C21, C21.0, C21.1, C21.2, C21.8], Malignant neoplasm of liver and intrahepatic bile ducts [C22, C22.0, C22.1, C22.2, C22.3, C22.4, C22.7, C22.9, 155], Malignant neoplasm of gallbladder [C23, 156], Malignant neoplasm of other and unspecified parts of biliary tract [C24, C24.0, C24.1, C24.8, C24.9], Malignant neoplasm of pancreas [C25, C25.0, C25.1, C25.2, C25.3, C25.4, C25.7, C25.8, C25.9, 157], Malignant neoplasm of other and ill-defined digestive organs [C26, C26.0, C26.1, C26.8, C26.9, 159], Malignant neoplasm of nasal cavity and middle ear [C30, C30.0, C30.1, 160], Malignant neoplasm of accessory sinuses [C31, C31.0, C31.1, C31.2, C31.3, C31.8, C31.9], Malignant neoplasm of larynx [C32, C32.0, C32.1, C32.2, C32.3, C32.8, C32.9, 161], Malignant neoplasm of trachea [C33], Malignant neoplasm of bronchus and lung [C34, C34.0, C34.1, C34.2, C34.3, C34.8, C34.9, 162], Malignant neoplasm of thymus [C37, 164], Malignant neoplasm of heart, mediastinum and pleura [C38, C38.0, C38.1, C38.2, C38.3, C38.4, C38,8, 163], | |

| | | | |
|---|---|---|---|
| | | Malignant neoplasm of other and ill-defined sites in the respiratory system and intrathoracic organs [C39, C39.0, C39.8, C39.9, 165], Malignant neoplasm of bone and articular cartilage of limbs [C40, C40.0, C40.1, C40.2, C40.3, C40.8, C40.9, 170], Malignant neoplasm of bone and articular cartilage of other and unspecified sites [C41, C41.0, C41.1, C41.2, C41.3, C41.4, C41.8, C41.9], Malignant melanoma of skin [C43, C43.0, C43.1, C43.2, C43.3, C43.4, C43.5, C43.6, C43.7, C43.8, C43.9, 172], Other malignant neoplasms of skin [C44, C44.0, C44.1, C44.2, C44.3, C44.4, C44.5, C44.6, C44.7, C44.8, C44.9, 173], Mesothelioma [C45, C45.0, C45.1, C45.2, C45.7, C45.9], Kaposi's sarcoma [C46, C46.0, C46.1, C46.2, C46.3, C46.7, C47.8, C46.9, 176], Malignant neoplasm of peripheral nerves and autonomic nervous system [C47, C47.0, C47.1, C47.2, C47.3, C47.4, C47.5, C47.6, C47.8, C47.9], Malignant neoplasm of retroperitoneum and peritoneum [C48, C48.0, C48.1, C48.2, C48.8, 158], Malignant neoplasm of other connective and soft tissue [C49, C49.0, C49.1, C49.2, C49.3, C49.4, C49.5, C49.6, C49.8, C49.9, 171], Malignant neoplasm of breast [C50, C50.0, C50.1, C50.2, C50.3, C50.4, C50.5, C50.6, C50.8, C50.9, 174, 175], Malignant neoplasm of vulva [C51, C51.0, C51.1, C51.2, C51.8, C51.9], Malignant neoplasm of vagina [C52], Malignant neoplasm of cervix uteri [C53, C53.0, C53.1, C53.8, C53.9, 180], Malignant neoplasm of corpus uteri [C54, C54.0, C54.1, C54.2, C54.3, C54.8, C54.9], Malignant neoplasm of uterus, part unspecified [C55, 179], Malignant neoplasm of ovary [C56, 183], Malignant neoplasm of other and unspecified female genital organs [C57, C57.0, C57.1, C57.2, C57.3, C57.4, C57.7, C57.8, C57.9, 184], Malignant neoplasm of placenta [C58, 181], Malignant neoplasm of penis [C60, C60.0, C60.1, C60.2, C60.8, C60.9, 187], Malignant neoplasm of prostate [C61, 185], Malignant neoplasm of testis [C62, C62.0, C62.1, | |

| | | | |
|---|---|---|---|
| | | C62.9, 186], Malignant neoplasm of other and unspecified male genital organs [C63, C63.0, C63.1, C63.2, C63.7, C63.8, C63.9], Malignant neoplasm of kidney, except renal pelvis [C64], Malignant neoplasm of renal pelvis [C65], Malignant neoplasm of ureter [C66], Malignant neoplasm of bladder [C67, C67.0, C67.1, C67.2, C67.3, C67.4, C67.5, C67.6, C67.7, C67.8, C67.9, 188], Malignant neoplasm of other and unspecified urinary organs [C68, C68.0, C68.1, C68.8, C68.9, 189], Malignant neoplasm of eye and adnexa [C69, C69.0, C69.1, C69.2, C69.3, C69.4, C69.5, C69.6, C69.8, C69.9, 190], Malignant neoplasm of meninges [C70, C70.0, C70.1, C70.9], Malignant neoplasm of brain [C71, C71.0, C71.1, C71.2, C71.3, C71.4, C71.5, C71.6, C71.7, C71.8, C71.9, 191], Malignant neoplasm of spinal cord, cranial nerves and other parts of central nervous system [C72, C72.0, C72.1, C72.2, C72.3, C72.4, C72.5, C72.8, C72.9, 192], Malignant neoplasm of thyroid gland [C73, 193], Malignant neoplasm of adrenal gland [C74, C74.0, C74.1, C74.9], Malignant neoplasm of other endocrine glands and related structures [C75, C75.0, C75.1, C75.2, C75.3, C75.4, C75.5, C75.8, C75.9, 194], Malignant neoplasm of other and ill-defined sites [C76, C76.0, C76.1, C76.2, C76.3, C76.4, C76.5, C76.7, C76.8, 195], Secondary and unspecified malignant neoplasm of lymph nodes [C77, C77.0, C77.1, C77.2, C77.3, C77.4, C77.5, C77.8, C77.9, 196], Secondary malignant neoplasm of respiratory and digestive organs [C78, C78.0, C78.1, C78.2, C78.3, C78.4, C78.5, C78.6, C78.7, C78.8, 197], Secondary malignant neoplasm of other sites [C79, C79.0, C79.1, C79.2, C79.3, C79.4, C79.5, C79.6, C79.7, C79.8, C79.9, 198], Malignant neoplasm without specification of site [C80, C80.0, C80.9, 199], Hodgkin lymphoma [C81, C81.0, C81.1, C81.2, C81.3, C81.4, C81.7, C81.9, 201], Follicular lymphoma [C82, C82.0, C82.1, C82.2, C82.3, C82.4, C82.5, C82.6, C82.7, C82.9], Non- | |

| | | | |
|---|---|---|---|
| | | follicular lymphoma [C83, C83.0, C83.1, C83.3, C83.5, C83.7, C83.8, C83.9], Mature T/NK-cell lymphomas [C84, C84.0, C84.1, C84.4, C84.5, C84.6, C84.7, C84.8, C84.9], Other and unspecified types of non-Hodgkin lymphoma [C85, C85.1, C85.2, C85.7, C85.9], Other specified types of T/NK-cell lymphoma [C86, C86.0, C86.1, C86.2, C86.3, C86.4, C86.5, C86.6], Malignant immunoproliferative diseases and certain other B-cell lymphomas [C88, C88.0, C88.2, C88.3, C88.4, C88.7, C88.9], Multiple myeloma and malignant plasma cell neoplasms [C90, C90.0, C90.1, C90.2, C90.3, 203], Lymphoid leukemia [C91, C91.0, C91.1, C91.3, C91.4, C91.5, C91.6, C91.7, C91.8, C91.9, 204], Myeloid leukemia [C92, C92.0, C92.1, C92.2, C92.3, C92.4, C92.5, C92.6, C92.7, C92.8, C92.9, 205], Monocytic leukemia [C93, C93.0, C93.1, C93.3, C93.7, C93.9, 206], Other leukemias of specified cell type [C94, C94.0, C94.2, C94.3, C94.4, C94.6, C94.7], Leukemia of unspecified cell type [C95, C95.0, C95.1, C95.7, C95.9, 208], Other and unspecified malignant neoplasms of lymphoid, hematopoietic and related tissue [C96, C96.0, C96.2, C96.4, C96.5, C96.6, C96.7, C96.8, C96.9, 202], Malignant neoplasms of independent (primary) multiple sites [C97] | |
| **Renal disease** | Renal/kidney failure [1192], Renal failure requiring dialysis [1193], Renal failure not requiring dialysis [1194], Diabetic nephropathy [1607], Nephritis [1608], Glomerulonephritis [1609] | Acute renal failure [N17.0, N17.1, N17.2, N17.8, N17.9], Chronic kidney disease [N18.1, N18.2, N18.3, N18.4, N18.5, N18.9, 585], Unspecified kidney failure [N19, 586], Unspecified renal sclerosis [587], Disorders resulting from impaired renal function [588] | Chronic renal failure [132033], Unspecified renal failure [132035], Chronic nephritic syndrome [132005], Nephrotic syndrome [132007] |
| **Connective-tissue disorder** | Connective tissue disorder [1373], Wegner's | Polyarthritis [M15.0, M15.1, M15.2, M15.3, M15.4, M15.8, M15.9], Coxarthrosis [M16.0, M16.1, M16.2, M16.3, M16.4, | Systemic lupus erythematosus [131895], Dermatopolymyositis |

| | | | |
|---|---|---|---|
| | granulomatosis [1378], Systemic lupus erythematosus [1381], Sjogren's syndrome/sicca syndrome [1382], Dermatopolymyositis [1383], Scleroderma/systemic sclerosis [1384], Polycystic kidney [1427], Dermatomyositis [1480] | M16.5, M16.6, M16.7, M16.9], Gonarthrosis [M17.0, M17.1, M17.2, M17.3, M17.4, M17.5, M17.9], Arthrosis of first carpometacarpal joint [M18.0, M18.1, M18.2, M18.3, M18.4, M18.5, M18.9], Osteoarthrosis and allied disorders [715] | [131897], Systemic sclerosis [131899], Seropositive rheumatoid arthritis [131849], Other rheumatoid arthritis [131851], Psoriatic and enteropathic arthropathies [131853] |
| **Blood-disorder** | Sickle cell disease [1139], Thalassaemia [1340], Haemochromatosis [1507] | | Thalassaemia [130632], Sickle-cell disorders [130635], Sarcoidosis [130686] |
| **Endocrine disorder** | | | Thyrotoxicosis [130701] |
| **Digestive disorder** | | | Crohn’s disease [131672], Ulcerative colitis [131629] |
| **Others** | Alcohol dependency [1408], Opioid dependency [1409] | | |

**Table-2. Cardiovascular Magnetic Resonance Imaging (MRI) phenotypes utilised to estimate the biological age of the cardiovascular system (*HeartAge*) in the UK-Biobank.**

| Abbreviations | MRI phenotypes | Orientation & type of images utilised |
|---|---|---|
| LV-EDV (ml) | Left ventricular end-diastolic volume | Short-axis stack cine images |
| LV-ESV (ml) | Left ventricular end-systolic volume | Short-axis stack cine images |
| LV-SV (ml) | Left ventricular stroke volume | Short-axis stack cine images |
| LV-EF (%) | Left ventricular ejection fraction | Short-axis stack cine images |
| LV-mass (g) | Left ventricular mass at end-diastole | Short-axis stack cine images |
| WT (mm) | Left ventricular wall thickness of the 16-AHA segments | Short-axis stack cine images |
| A-WT (mm) | Average left ventricular wall thickness | Short-axis stack cine images |
| RV-EDV (ml) | Right ventricular end-diastolic volume | Short-axis stack cine images |
| RV-ESV (ml) | Right ventricular end-systolic volume | Short-axis stack cine images |
| RV-SV (ml) | Right ventricular stroke volume | Short-axis stack cine images |
| RV-EF (%) | Right ventricular ejection fraction | Short-axis stack cine images |
| LA-res (ml) | Left atrial reservoir volume at ventricular end-systole | Four/two-chamber cine images |
| LA-cond (ml) | Left atrial conduit volume immediately before atrial contraction | Four/two-chamber cine images |
| LA-pump (ml) | Left atrial pump volume immediately after the atrial contraction | Four/two-chamber cine images |
| RA-res (ml) | Right atrial reservoir volume at ventricular end-systole | Four-chamber cine images |
| RA-cond (ml) | Right atrial conduit volume immediately before atrial contraction | Four-chamber cine images |

| | | |
|---|---|---|
| RA-pump (ml) | Left atrial pump volume immediately after the atrial contraction | Four-chamber cine images |
| AoED (mm2) | Ascending aorta cross-sectional area at end-diastole | Trans-axial cine images |
| AoES (mm2) | Ascending aorta cross-sectional area at end-systole | Trans-axial cine images |
| AoD ($10^{-3}$ mm $Hg^{-1}$) | Aortic distensibility of the ascending aorta | Derived measure (reference 1) |

**Table-3. Diagnostic codes used for the composite cardiovascular outcome and all-cause mortality.**

Cumulative death and adverse cardiovascular events were identified through ICD-9, ICD-10 and specific UK-Biobank categories / data-fields following the date of cardiovascular magnetic resonance imaging (UK-Biobank data-field 53, instance 2).

| | **Death registry** | **Hospital episode statistics** | **First occurrence** |
|---|---|---|---|
| **All-cause mortality (n=447)** | 40000 | | |
| **Heart Failure (n=443)** | | Heart failure [I50, I50.1, I50.9] | Heart failure [131355], Pulmonary oedema [131525] |
| **Ischemic heart diseases (n=1170)** | | Acute myocardial infarction [I21, I21.0, I21.1, I21.2, I21.3, I21.4, I21.9], Subsequent ST elevation and non ST elevation myocardial infarction [I22, I22.0, I22.1, I22.2, I22.8, I22.9], Complications following STEMI and NSTEMI myocardial infarction [I23, I23.0, I23.1, I23.2, I23.3, I23.4, I23.5, I23.6, I23.8], Dressler's syndrome [I24.1], Old myocardial infarction [I25.2] | Angina pectoris [131297], Acute myocardial infarction [131299], Subsequent myocardial infarction [131301], Complications following acute myocardial infarction [131303], Other acute ischaemic heart diseases [131305], Chronic ischaemic heart disease [131307] |
| **Ischemic stroke (n=154)** | | Cerebral infarction [I63, I63.0, I63.1, I63.2, I63.3, I63.4, I63.5, I63.6, I63.8, I63.9], Stroke, not specified as haemorrhage or infarction [I64] | |

| | | | |
|---|---|---|---|
| **Cardiac rhythm abnormalities (n=1647; 1482 [90%] captured by hospital episode statistics)** | | Atrioventricular and left bundle-branch block [I44], Other conduction disorder [I45, I45.0, I45.1, I45.2, I45.3, I45.4, I45.5], Atrial fibrillation and flutter [I48] | Atrioventricular and left bundle-branch block [131343], Other conduction disorders [131345], Atrial fibrillation and flutter [131351], Other cardiac arrhythmias [131353] |

**Table-4. Cardiovascular Magnetic Resonance Imaging (MRI) phenotypes utilised to measure the biological age of the cardiovascular system in the Multi-Ethnic Study of Atherosclerosis (MESA) cohort (*HeartAge*(mesa)).**

| Abbreviations | MRI phenotypes | Orientation & type of images utilised |
|---|---|---|
| LV-EDV (ml) | Left ventricular end-diastolic volume | Short-axis stack cine images |
| LV-ESV (ml) | Left ventricular end-systolic volume | Short-axis stack cine images |
| LV-SV (ml) | Left ventricular stroke volume | Short-axis stack cine images |
| LV-mass (g) | Left ventricular mass at end-diastole | Short-axis stack cine images |
| RV-EDV (ml) | Right ventricular end-diastolic volume | Short-axis stack cine images |
| RV-ESV (ml) | Right ventricular end-systolic volume | Short-axis stack cine images |
| RV-SV (ml) | Right ventricular stroke volume | Short-axis stack cine images |
| RV-EF (%) | Right ventricular stroke volume | Short-axis stack cine images |
| RV-mass (g) | Right ventricular mass at end-diastole | Short-axis stack cine images |
| LA-res (ml) | Left atrial reservoir volume at ventricular end-systole | Four/two-chamber cine images |
| LA-cond (ml) | Left atrial conduit volume immediately before atrial contraction | Four/two-chamber cine images |
| LA-pump (ml) | Left atrial pump volume immediately after the atrial contraction | Four/two-chamber cine images |
| RA-res (ml) | Right atrial reservoir volume at ventricular end-systole | Four-chamber cine images |
| RA-cond (ml) | Right atrial conduit volume immediately before atrial contraction | Four-chamber cine images |
| RA-pump (ml) | Left atrial pump volume immediately after the atrial contraction | Four-chamber cine images |

| | | |
|---|---|---|
| AoED (mm2) | Ascending aorta cross-sectional area at end-diastole | Trans-axial cine images |
| AoES (mm2) | Ascending aorta cross-sectional area at end-systole | Trans-axial cine images |
| AoD ($10^{-3}$ mm $Hg^{-1}$) | Aortic distensibility of the ascending aorta | Derive measure (reference 1) |

**Table-5. Cox regression proportional hazard-ratio models for the composite cardiovascular outcome and its individual components in the UK-Biobank**

| | **Female** | | | **Male** | | |
|---|---|---|---|---|---|---|
| ***Fully adjusted Cox regression proportional hazard-ratio models*** | | | | | | |
| **Composite Outcome** | **HR** | **95%CI** | **P-Value** | **HR** | **95%CI** | **P-Value** |
| *HeartAge-gap*, yrs | 1.028 | 1.007-1.049 | 0.008 | 1.016 | 1.001-1.031 | 0.034 |
| Chronological age, yrs | 1.067 | 1.056 -1.079 | <0.001 | 1.070 | 1.061-1.077 | <0.001 |
| Body-mass-index (kg/m2) | 1.023 | 1.007-1.040 | 0.006 | 1.048 | 1.034-1.062 | <0.001 |
| Hypertension | 1.486 | 1.270-1.737 | <0.001 | 1.211 | 1.084-1.354 | 0.001 |
| Diabetes | 1.352 | 1.022-1.791 | 0.035 | 1.208 | 1.026-1.423 | 0.023 |
| Ischaemic heart disease | 3.356 | 2.708-4.158 | <0.001 | 2.088 | 1.826-2.389 | <0.001 |
| Mean blood pressure (mmHg) | 1.001 | 0.998-1.004 | 0.500 | 1.004 | 1.002-1.007 | 0.002 |

Mean blood pressure is calculated by the following formula: brachial diastolic blood pressure + 1/3 x (brachial systolic blood pressure – brachial diastolic blood pressure).

Brachial systolic and diastolic blood pressures were measured at the time of MRI.

Abbreviations: CI: Confidence interval; HR: Hazard Ratio.

**Table-6. Baseline characteristics of the Multi-Ethnic Study of Atherosclerosis cohort**

| | **Females (n=472)** | **Males (n=422)** |
|---|---|---|
| **Baseline characteristics** | | |
| Age (years) | 61.30±10.14 | 59.80±9.87 |
| Body-mass-index (kg/m$^2$) | 28.74±5.56 | 27.53±3.85 |
| Systolic blood pressure (mmHg) | 136.13±21.59 | 135.05±18.4 |
| Diastolic blood pressure (mmHg) | 73.46±10.80 | 80.51±10.34 |
| Active smoking, n (%) | 60 (12.7%) | 61 (14.4%) |
| Diabetes or impaired fasting glucose, n (%) | 118 (25.1%) | 133 (31.5%) |
| Hypertension, n (%) | 204 (43.2%) | 164 (38.9%) |
| Dyslipidemia, n (%) | 172 (36.4%) | 123 (29.1%) |
| White/Black/Hispanic-Latino/Chinese Ethnicity, n | 184/132/103/53 | 162/127/80/53 |
| **MRI Phenotypes** | | |
| LV end-diastolic volume (ml) | 114.87±24.23 | 142.77±28.75 |
| LV end-systolic volume (ml) | 33.42±12.71 | 47.86±15.05 |
| LV stroke volume (ml) | 81.45±16.17 | 94.91±19.00 |
| LV ejection fraction (%) | 71.29±6.51 | 66.83±6.49 |
| LV end-diastolic mass (g) | 125.68±27.12 | 169.88±32.16 |
| RV end-diastolic volume (ml) | 110.11±23.37 | 144.28±28.95 |
| RV end-systolic volume (ml) | 31.02±10.36 | 46.39±13.99 |
| RV stroke volume (%) | 79.09±16.84 | 97.89±20.33 |

| | | |
|---|---|---|
| RV end-diastolic mass (g) | 19.29±3.78 | 23.56±4.28 |
| RV ejection fraction (%) | 72.09±6.02 | 68.05±6.13 |
| RA reservoir (ml) | 38.31±12.60 | 46.04±16.44 |
| RA conduit volume (ml) | 30.38±6.84 | 36.70±14.73 |
| RA pump volume (ml) | 20.02±8.55 | 25.06±12.15 |
| LA reservoir volume (ml) | 54.13±17.35 | 59.16±19.25 |
| LA conduit volume (ml) | 42.52±15.08 | 48.20±16.66 |
| LA pump volume (ml) | 28.03±11.50 | 31.77±12.10 |
| Ascending aortic area at end-diastole ($cm^2$) | 7.01±1.56 | 8.12±1.70 |
| Ascending aortic area at end-systole ($cm^2$) | 7.62±1.58 | 8.83±1.73 |
| Ascending aortic distensibility ($10^{-3}mmHg^{-1}$) | 0.002±0.001 | 0.002±0.001 |

Atrial volumes: LA volumes were derived from bi-planar analysis of 2- and 4-chamber cine images, and RA volumes from 4-chamber cine only. Volumes were sampled at: (i) ventricular end-systole (maximum atrial volume, reservoir), (ii) ventricular diastole immediately before atrial contraction (conduit), and (iii) ventricular end-diastole after atrial contraction (minimum atrial volume, pump). Accordingly, reservoir volume = maximal atrial volume, conduit volume = atrial volume pre-atrial contraction, and pump volume = minimal atrial volume (1,2).

Ascending Aortic Distensibility: The cross-sectional area of the ascending aorta was extracted across the cardiac cycle, with minimum (Amin) and maximum (Amax) areas identified. Distensibility was computed as (Amax – Amin) / (Amin × PP), where PP denotes central pulse pressure in mmHg measured at the time of MRI (3).

Abbreviations: LA: Left atrium; LV: Left ventricular; MRI: Magnetic Resonance Imaging; RA: Right atrium; RV: Right ventricular.

**Table-7. The Cox regression proportional hazard-ratio models for the hard cardiovascular outcome in the Multi-Ethnic Study of Atherosclerosis (MESA) cohort**

| Outcome | Female | | | Male | | |
|---|---|---|---|---|---|---|
| | **HR** | **95%CI** | **P-Value** | **HR** | **95%CI** | **P-Value** |
| *HeartAge-gap*(mesa) | 1.113 | 1.025-1.210 | 0.011 | 1.026 | 0.959-1.097 | 0.458 |
| Chronological age | 1.083 | 1.043-1.124 | <0.001 | 1.044 | 1.014-1.075 | 0.004 |
| Body-mass-index | 1.040 | 0.977-1.107 | 0.221 | 0.988 | 0.920-1.060 | 0.728 |
| Hypertension | 1.186 | 0.600-2.343 | 0.623 | 2.293 | 1.274-4.124 | 0.006 |
| Diabetes | 1.307 | 0.648-2.633 | 0.454 | 1.631 | 0.944-2.818 | 0.079 |
| Abbreviations: CI: Confidence interval; HR: Hazard ratio | | | | | | |

## Supplemental Figures

### Figure-1. SHAP values for the main features of the XGBoost model in the UK-Biobank.

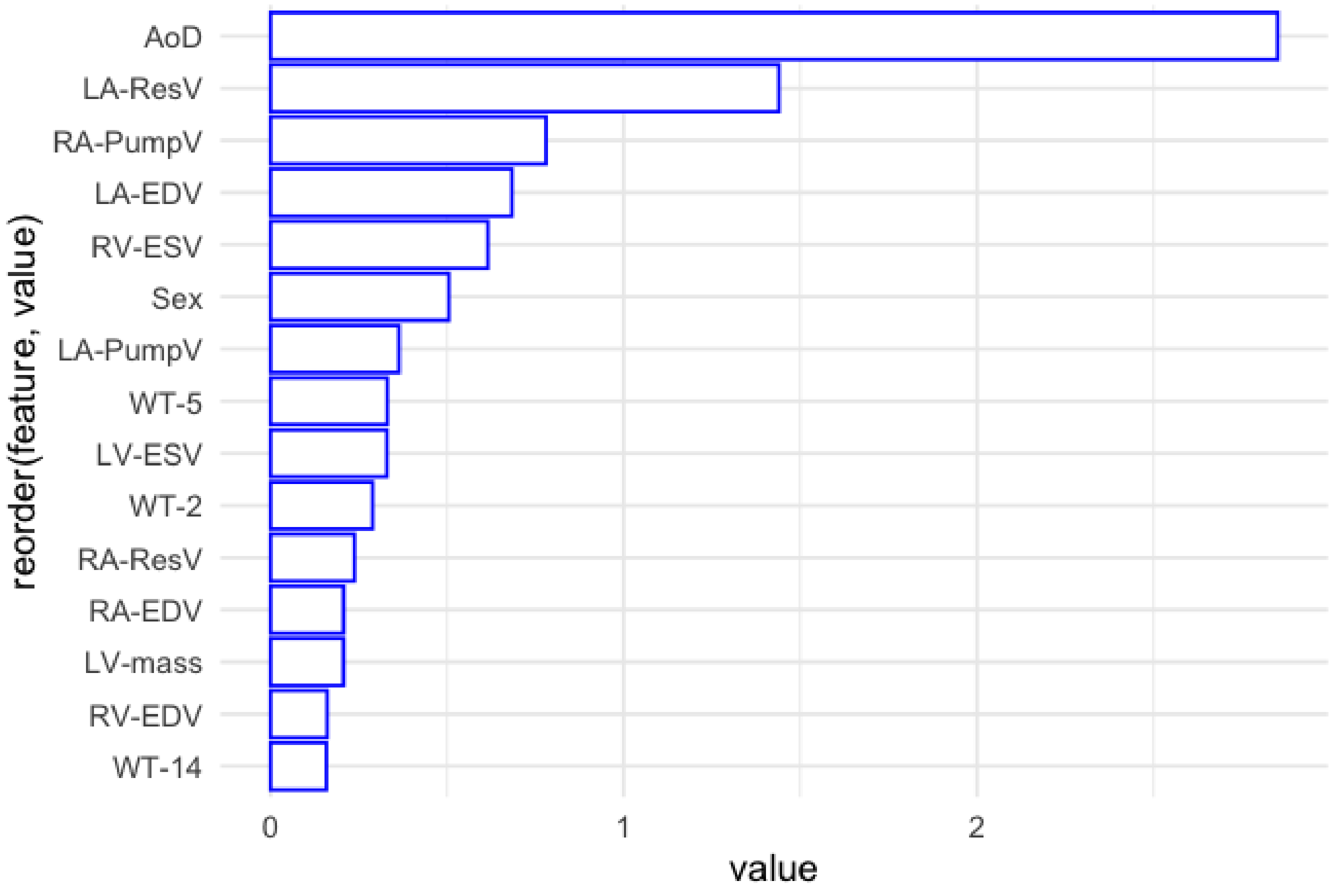


Abbreviations: AoD: Aortic distensibility; EDV: End-diastolic volume; ESV: End-systolic volume; LA: Left atrium; LV: Left ventricle; PumpV: Pump volume; RA: Right atrium; ResV: Residual volume; RV: Right ventricle; SHAP: SHapley Additive exPlanations; V: Volume (for atria); WT: Wall thickness.

**Figure-2. Distribution of *HeartAge*(mesa), *HeartAge-gap*(mesa) and chronological age in the Multi-Ethnic Study of Atherosclerosis (MESA) cohort**.

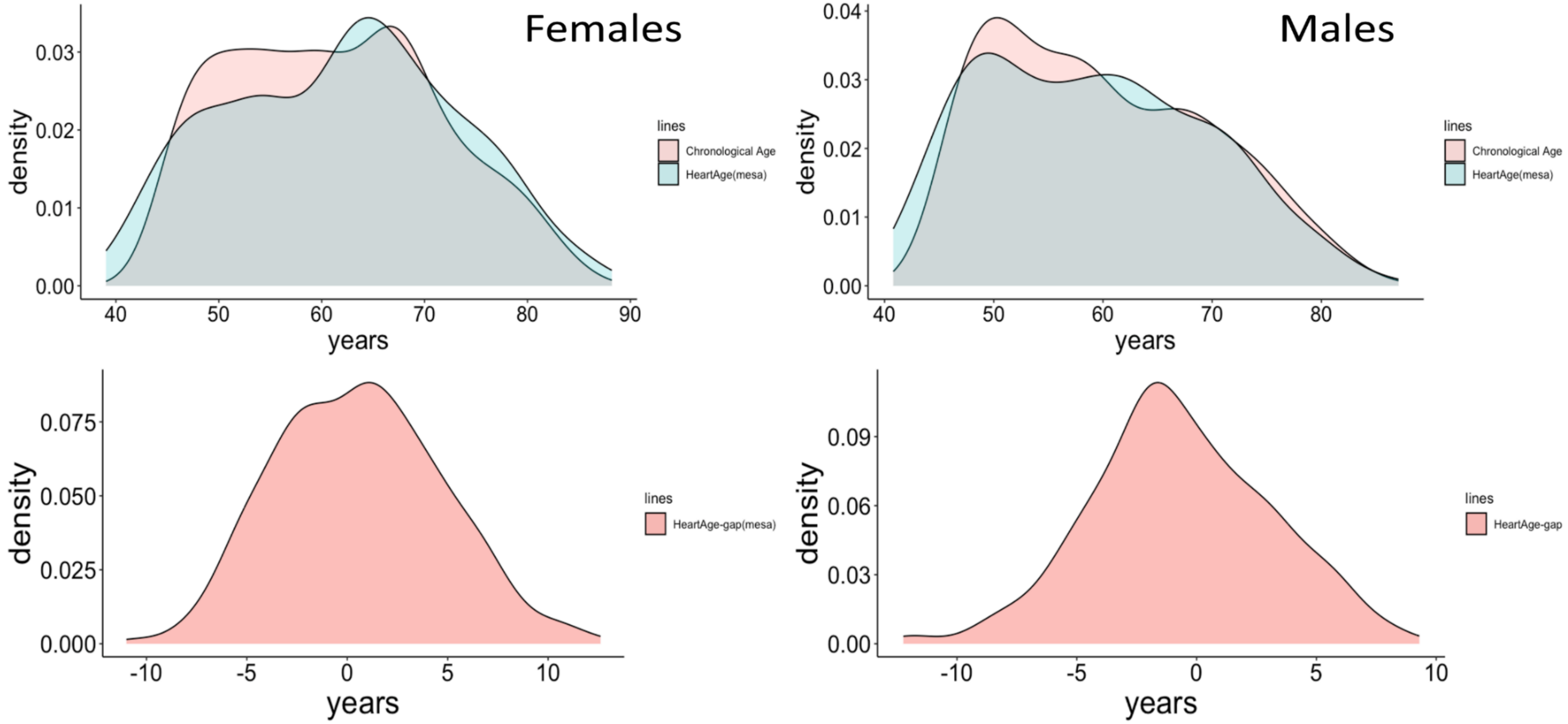


Upper panel: Density plots showing the distribution of chronological age, *HeartAge* (cardiovascular biological age estimate) in female and male participants.

Bottom panel: Density plots showing the distribution of *HeartAge-gap* (difference between cardiovascular biological age estimate and chronological age) in female and male participants.

# References

1. Ruijsink B, Puyol-Antón E, Oksuz I, Sinclair M, Bai W, Schnabel JA, et al. Fully Automated, Quality-Controlled Cardiac Analysis From CMR: Validation and Large-Scale Application to Characterize Cardiac Function. JACC Cardiovasc Imaging. 2020 Mar;13(3):684–95.

2. Mariscal-Harana J, Asher C, Vergani V, Rizvi M, Keehn L, Kim RJ, et al. An artificial intelligence tool for automated analysis of large-scale unstructured clinical cine cardiac magnetic resonance databases. Eur Heart J - Digit Health. 2023 Oct 1;4(5):370–83

3. Cecelja M, Ruijsink B, Puyol-Antón E, Li Y, Godwin H, King AP, Razavi R, Chowienczyk P. Aortic Distensibility Measured by Automated Analysis of Magnetic Resonance Imaging Predicts Adverse Cardiovascular Events in UK Biobank. J Am Heart Assoc. 2022 Dec 6;11(23):e026361. doi: 10.1161/JAHA.122.026361
